# Annotation, phylogeny and expression analysis of the nuclear factor Y gene families in common bean (*Phaseolus vulgaris*)

**DOI:** 10.3389/fpls.2014.00761

**Published:** 2015-01-14

**Authors:** Carolina Rípodas, Mélisse Castaingts, Joaquín Clúa, Flavio Blanco, María Eugenia Zanetti

**Affiliations:** Instituto de Biotecnología y Biología Molecular, Universidad Nacional de La Plata, CCT-CONICETLa Plata, Argentina

**Keywords:** CCAAT box, gene regulation, legumes, nodulation, transcription factors, symbiosis

## Abstract

In the past decade, plant nuclear factor Y (NF-Y) genes have gained major interest due to their roles in many biological processes in plant development or adaptation to environmental conditions, particularly in the root nodule symbiosis established between legume plants and nitrogen fixing bacteria. NF-Ys are heterotrimeric transcriptional complexes composed of three subunits, NF-YA, NF-YB, and NF-YC, which bind with high affinity and specificity to the CCAAT box, a *cis* element present in many eukaryotic promoters. In plants, NF-Y subunits consist of gene families with about 10 members each. In this study, we have identified and characterized the NF-Y gene families of common bean (*Phaseolus vulgaris*), a grain legume of worldwide economical importance and the main source of dietary protein of developing countries. Expression analysis showed that some members of each family are up-regulated at early or late stages of the nitrogen fixing symbiotic interaction with its partner *Rhizobium etli*. We also showed that some genes are differentially accumulated in response to inoculation with high or less efficient *R. etli* strains, constituting excellent candidates to participate in the strain-specific response during symbiosis. Genes of the NF-YA family exhibit a highly structured intron-exon organization. Moreover, this family is characterized by the presence of upstream ORFs when introns in the 5′ UTR are retained and miRNA target sites in their 3′ UTR, suggesting that these genes might be subjected to a complex post-transcriptional regulation. Multiple protein alignments indicated the presence of highly conserved domains in each of the NF-Y families, presumably involved in subunit interactions and DNA binding. The analysis presented here constitutes a starting point to understand the regulation and biological function of individual members of the NF-Y families in different developmental processes in this grain legume.

## Introduction

Nuclear factor Ys (NF-Ys) are heterotrimeric transcription factors evolutionary conserved in yeast, mammals and plants that have emerged as important regulators of gene expression. The complex is composed of three subunits: NF-YA, NF-YB, and NF-YC (also known as HAP2, HAP3, and HAP5, respectively). The current model for assembling of the NF-Y complex in mammals involves the dimerization of the NF-YB and NF-YC subunits through the interaction of their histone-fold domains (HFDs) in the cytoplasm, the translocation of the heterodimer to the nucleus and the association with the NF-YA subunit to form the mature and functional complex (Kahle et al., 2005). This trimer recognizes and binds CCAAT boxes, *cis* elements present in many eukaryotic promoters, activating or repressing transcription of the downstream genes (Ceribelli et al., 2008).

In yeast and metazoan, each NF-Y subunit is encoded by one or two genes, whereas plant families have largely expanded (Petroni et al., 2012; Laloum et al., 2013). The *Arabidopsis thaliana* genome encodes 10 NF-YA, 10 NF-YB, and 10 NF-YC subunits, whereas in rice (*Oryza sativa*) these families have 10, 11, and 7 genes, respectively (Siefers et al., 2009; Petroni et al., 2012; Laloum et al., 2013). The analysis of these families in leguminous plants identified 8, 19, and 11 members for the NF-YA, -YB, and -YC families, respectively, in the model *Medicago truncatula*, whereas in soybean (*Glycine max*) these families have expanded to 20, 39, and 27 members for each subunit, respectively (Laloum et al., 2013). However, the annotation of *M. truncatula* and soybean included two groups of more divergent HFD containing proteins (referred to as NC2β and NC2α) within the NF-YB and -YC families, respectively.

The expansion of the NF-Y gene family in the plant lineage could result theoretically in more than a thousand alternative unique heterotrimeric combinations (Siefers et al., 2009). This has led to the speculation that this combinatorial association between NF-Y subunits may provide a versatile and flexible regulatory system that integrates developmental and environmental inputs to fine-tune transcriptional outputs. Two systematic studies analyzed the interactions between Arabidopsis NF-Y subunits using the yeast two hybrid system (Calvenzani et al., 2012; Hackenberg et al., 2012), leading to the conclusion that most NF-YB and NF-YC subunits can interact promiscuously with each other, and that heterodimer formation trough the HFD seems to be a prerequisite for translocation into the nucleus, association with the NF-YA subunit and the subsequent binding to DNA.

The function of the NF-Y complex has been extensively characterized in mammals, where it plays a central role in the control of cell proliferation and early stages of development. In plants, forward and reverse genetic approaches have led to the identification of several genes encoding NF-Y subunits that are required for the correct development of plant programs such as embryogenesis (Lotan et al., 1998; Kwong et al., 2003), seed germination (Warpeha et al., 2007; Kumimoto et al., 2013), chloroplast biogenesis (Miyoshi et al., 2003), flowering (Ben-Naim et al., 2006; Kumimoto et al., 2010; Tiwari et al., 2010) and root elongation (Ballif et al., 2011). Genes encoding NF-Y subunits have been implicated also in the organogenesis and development of symbiotic root nodules in leguminous plants, where they act as components of a hierarchical transcriptional activation cascade in the nodulation signaling pathway (Combier et al., 2006; Zanetti et al., 2010; Soyano et al., 2013; Laloum et al., 2014; Laporte et al., 2014).

Expression analyses of individual members of each NF-Y family conducted in *Arabidopsis*, rice, wheat (*Triticum aestivum*), *Brachypodium distachion* and canola (*Brassica napus* L.) have revealed organ-specific expression patterns for several members of each subunit family (Gusmaroli et al., 2002; Stephenson et al., 2007; Thirumurugan et al., 2008; Siefers et al., 2009; Cao et al., 2011; Liang et al., 2014). In legumes, expression analyses have been conducted for individual NF-Y members at different stages of nodule formation or during arbuscular mycorrhization (Combier et al., 2006, 2008; Zanetti et al., 2010; Soyano et al., 2013; Laloum et al., 2014; Laporte et al., 2014; Hogekamp et al., 2011; Schaarschmidt et al., 2013). However, a systematic analysis of gene expression for each member of individual subunits in a single legume has not been reported.

Common bean is the most important grain legume for human consumption, representing the major source of proteins and essential nutrients in the diets of developing countries. This species is original of America, where two main centers of genetic diversification have been proposed: the Mesoamerican and the South Andean centers (Gepts, 1998). As other legumes, common bean establishes a nitrogen-fixing symbiotic interaction with soil bacteria collectively known as rhizobia. This interaction results in the formation of a new organ in the root, the nodule, which allocates bacteria and provides the appropriate environment for nitrogen fixation. Among the several *Rhizobium* species recognized as microsymbionts of common bean, *R. etli* is the predominant species associated to nodules of wild and domesticated common bean plants in America (Aguilar et al., 1998). Moreover, its has been shown that Mesoamerican accessions of common bean show a preferential and more efficient nodulation with *R. etli* strains that are predominant in Mesoamerican soils respect to those that are more abundant in Andean soils (Aguilar et al., 2004; Peltzer Meschini et al., 2008; Mazziotta et al., 2013). A member of the NF-YC family, named PvNF-YC1, was shown to play a role in nodule organogenesis and rhizobial infection, as well as in the preferential and more efficient association observed between Mesoamerican accession and sympatric rhizobial strains (Zanetti et al., 2010). Moreover, PvNF-YC1 physically interacts (in yeast and *in planta*) with another transcription factor belonging to the GRAS family, which also plays a role in nodule development and in the progression of the infection process in the common bean-*R. etli* interaction (Battaglia et al., 2014; Rípodas et al., 2014).

Considering the important role played by NF-Y genes in root nodule symbiosis, we took advantage of the recently released genome of common bean v1.0 (Schmutz et al., 2014) to annotate and analyze the gene families of each NF-Y subunit in this grain legume. The analysis identified 9, 14, and 7 members of the NF-YA, NF-YB, and NF-YC gene families, respectively. Genes of the NF-YA family are highly structured, showing a complex array of exons and introns in the coding and untranslated regions, a feature that was not observed in the NF-YB and NF-YC families. The quantitative analysis of the expression allowed us to identify transcripts that accumulate at high levels in nodules or in roots at early time points after inoculation with rhizobia, whereas some others exhibit specific expression in aerial tissues. In addition, the use of *R. etli* strains with different degrees of nodulation efficiency led us to identify some NF-Y members whose mRNA accumulates at higher levels in either the more or the less efficient symbiotic interactions.

## Materials and methods

### Sequence analysis, chromosome mapping, alignments and phylogenetic trees

Individual subunits were identified by TBLASTN searches against the common bean genome V1.0 database (http://phytozome.jgi.doe.gov) using the full length amino acid sequences of the 30 members of Arabidopsis NF-Y gene family. The physical position of PvNF-Y members was mapped on common bean chromosomes using Map-view function available at Plant Genome Duplication Database (PGDD) as implemented by Lee et al. (2013). Alignments and phylogenies were created from full-length protein sequences. *Arabidopsis thaliana, Medicago truncatula* and *Glycine max* sequences were obtained from the GenBank, TAIR or Phytozome v10 websites. Multiple sequence alignments were performed with ClustalW (Thompson et al., 1994) and shaded with BOXSHADE 3.21 software (http://www.ch.embnet.org/software/BOX_form.html). Protein alignments were imported into MEGA5, where rooted phylogenetic trees were created (Tamura et al., 2011). The locus search function of PGDD was used to identify syntenic regions (colinear blocks) containing PvNF-Y loci using default parameters (Lee et al., 2013). miR169 target sites in NF-YA family members were identified with Target-align (Xie and Zhang, 2010) using default parameters. The presence of uORF in *P. vulgaris* NF-YA genes was analyzed using Vector NTI (Invitrogen, www.lifetechnolgies.com), allowing a minimum length of 60 nt.

### Plant growth and rhizobial inoculation

Seeds of common bean (NAG12 accession) were surface sterilized and germinated on wet paper for 2–3 days at 26°C in the dark as previously described (Peltzer Meschini et al., 2008). Germinated seedlings were transferred to acrylic boxes containing slanted agar-Fahraeus media free of nitrogen (Fahraeus, 1957). Seedlings were grown in a MLR-350HT growth chamber (Sanyo Electric Co., www.panasonic.net/sanyo) maintained at 26°C with a 16/8 h day/night cycle and 80% humidity. Seven days after transplantation to acrylic boxes, leaf, stem and root tissue was collected. For nodulation, roots of 7 day-old seedlings were inoculated with 5 ml of a culture of *R. etli* strain SC15 or 55N1 grown in liquid YEM media until the OD_600_ reached 0.8. Nodules were collected at 7 and 14 days post inoculation (dpi). In all cases, tissue from 3 plants was pooled for each biological replicate, frozen in liquid N_2_ and stored at −80°C.

### RNA extraction and RT-qPCR

Total RNA extraction was performed with Trizol following manufacture's instructions (Invitrogen). RNA concentration was determined by measuring absorbance at 260 nm in a Nanodrop ND-1000 (Nanodrop Technologies Inc., www.nanodrop.com) and quality was evaluated using the Agilent RNA 6000 nano kit and the Agilent Technologies 2100 Bioanalyzer (www.agilent.com). One μg of total RNA was treated with RNAse free-DNAse I and used in a reverse transcription reaction with 0.5 μg of oligo dT_15_ and 200 units of M-MLV-RT following instructions provided by the manufacturer (Promega, www.promega.com). Individual cDNA samples were used in quantitative PCR (qPCR) reactions as previously reported (Zanetti et al., 2010) with gene specific primers (see Table S1). Amplification of common bean elongation factor 1α (*EF1α*) was used to normalize the amount of template cDNA as reported (Peltzer Meschini et al., 2008). Statistical significance of differences between samples was determined by unpaired two tailed *t*-tests using at least two biological replicates.

## Results

### Identification, annotation, and structure of *P. vulgaris* NF-Y genes

**Figure 1:**
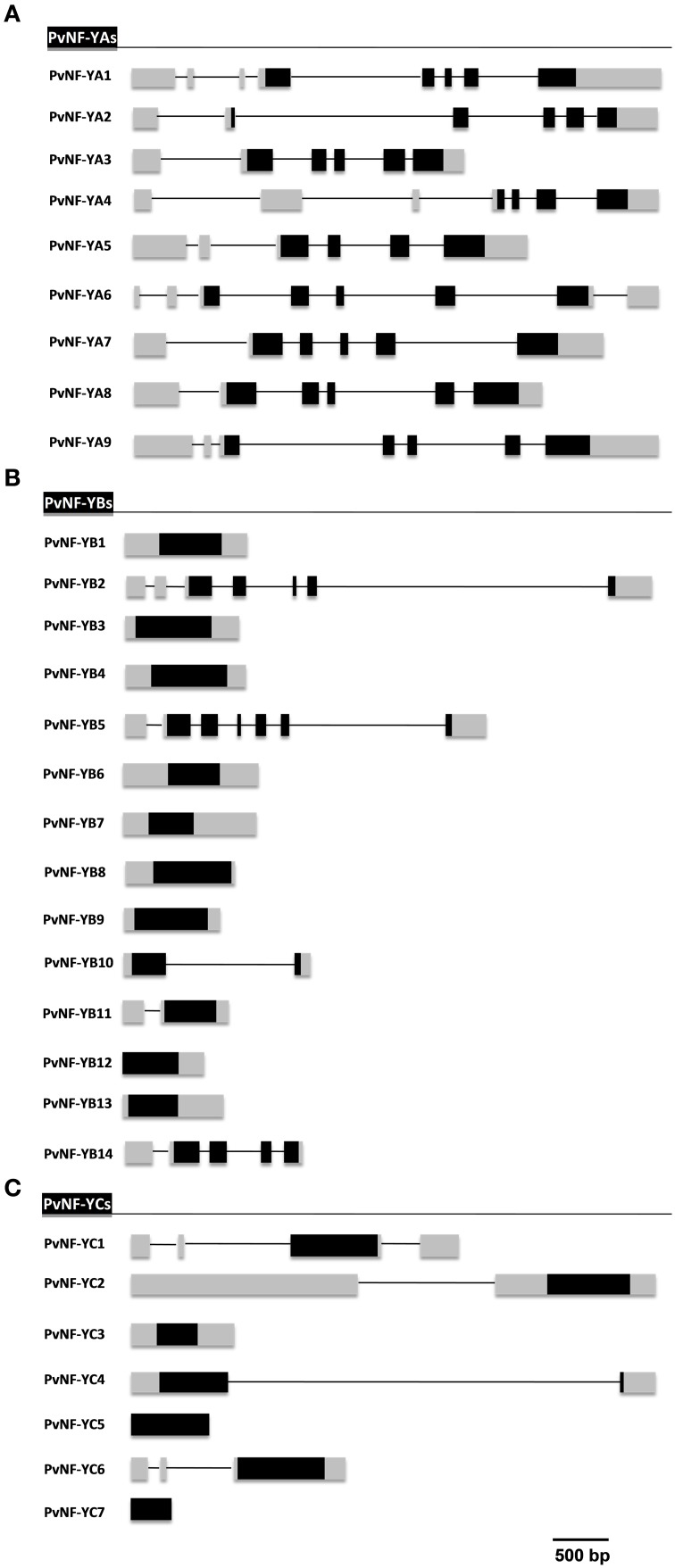
**Gene structure of *PvNF-Y* subunits family members in *P. vulgaris***. Scheme of intron/exon organization of gene members of *PvNF-YA* **(A)**, *PvNF-YB* **(B)**, and *PvNF-YC* **(C)** families predicted from the gene models and RNA sequencing studies available at the *P. vulgaris* genome V1.0 database. Lines represent introns and boxes represent exons. Gray boxes correspond to 5′ and 3′ untranslated regions (UTR) and black boxes correspond to coding sequence (CDS) regions. The sizes of intron and exon can be estimated using the reference scale bar of 500 bp. Note that *PvNF-YB12* does not have a 5′ UTR annotated in the common bean genome. Similarly, that *PvNF-YC5* and *-YC7* do not have either 5′ or 3′ UTRs annotated in the common bean genome.

We used the full length amino acid sequences of the 30 members of Arabidopsis NF-Y gene family to sequentially BLAST search the *P. vulgaris* genome V1.0 database (publicly available at http://phytozome.jgi.doe.gov) using an *E*-value *cutoff* of 10^−5^. We identified 30 members of these gene families distributed as 9 NF-YAs, 14 NF-YBs, and 7 NF-YCs (Table 1). Based on the recent recommendations to assign names for plant NF-Y families (Laloum et al., 2013), we have designated each member with a two letter code corresponding to the species initials (Pv for *P. vulgaris*) followed by NF-Y, the letter for each subunit (A, B, or C) and finally by a number. Because there is no previous characterization of any member of the NF-YB family in common bean, there is not a previous scheme to number members of this family. In the cases of NF-YA and NF-YC families, PvNF-YA1 and PvNF-YC1, -YC2, and -YC3 were previously characterized (Zanetti et al., 2010; Battaglia et al., 2014); therefore, we continued numbering after these genes. Table 1 presents assigned names together with the genomic gene ID and its best homologs/putative orthologs in *Arabidopsis*. Table S2 presents, in addition, the best homologs/putative orthologs in two other legume species (*M. truncatula* and soybean). The gene structure of each NF-Y gene predicted from the available gene models are presented in Figure 1. The physical position of the 30 PvNF-Y members was mapped on the chromosomes available from the common bean genome. PvNF-Y genes were distributed on 10 out of the 11 common bean chromosomes (Figure S1). In particular, the 9 PvNF-YA genes were uniformly distributed on several chromosomes, whereas members of the PvNF-YB family showed a higher degree of association, with five members located on chromosome Pv Chr07. Members of the PvNF-YC family mapped only in five chromosomes, with one or two genes per chromosome.

**Table 1:** **Annotation of common bean NF-Y families**.

***PvNF-YA* family**			***PvNF-YB* family**				***PvNF-YC* family**	
**Name**	**Locus name**	**Best *At* homolog**	**Name**	**Locus name**	**Best *At* homolog**	**Name**	**Locus name**	**Best *At* homolog**
PvNF-YA1	Phvul.001G196800	NF-YA10 (AT5G06510)	PvNF-YB1	Phvul.008G278900	NF-YB3 (AT4G14540)	PvNF-YC1	Phvul.006G093200	NF-YC9 (AT1G08970)
PvNF-YA2	Phvul.002G246600	NF-YA7 (AT1G30500)	PvNF-YB2	Phvul.007G134000	NF-YB8 (AT2G37060)	PvNF-YC2	Phvul.005G050000	NF-YC2 (AT1G56170)
PvNF-YA3	Phvul.005G156100	NF-YA8 (AT1G17590)	PvNF-YB3	Phvul.006G139400	NF-YB3 (AT4G14540)	PvNF-YC3	Phvul.010G102300	NF-YC4 (AT5G63470)
PvNF-YA4	Phvul.003G133100	NF-YA3 (AT1G72830)	PvNF-YB4	Phvul.002G264300	NF-YB3 (AT4G14540)	PvNF-YC4	Phvul.009G166000	NF-YC1 (AT3G48590)
PvNF-YA5	Phvul.008G283100	NF-YA9 (AT3G20910)	PvNF-YB5	Phvul.007G163100	NF-YB10 (AT3G53340)	PvNF-YC5	Phvul.006G152400	NF-YC2 (AT1G56170)
PvNF-YA6	Phvul.011G211300	NF-YA9 (AT3G20910)	PvNF-YB6	Phvul.007G165100	NF-YB5 (AT2G47810	PvNF-YC6	Phvul.007G181400	NF-YC9 (AT1G08970)
PvNF-YA7	Phvul.006G062200	NF-YA1 (AT5G12840)	PvNF-YB7	Phvul.003G086800	NF-YB3 (AT4G14540)	PvNF-YC7	Phvul.010G082500	NF-YC3 (AT1G54830)
PvNF-YA8	Phvul.010G133300	NF-YA3 (AT1G72830)	PvNF-YB8	Phvul.002G273800	NF-YB7 (AT2G13570)			
PvNF-YA9	Phvul.007G267100	NF-YA10 (AT5G06510)	PvNF-YB9	Phvul.009G155500	NF-YB7 (AT2G13570)			
			PvNF-YB10	Phvul.009G246400	NF-YB5 (AT2G47810)			
			PvNF-YB11	Phvul.003G103500	NF-YB5 (AT2G47810)			
			PvNF-YB12	Phvul.007G234800	NF-YB6 (AT5G47670)			
			PvNF-YB13	Phvul.007G195900	NF-YB4 (AT1G09030)			
			PvNF-YB14	Phvul.001G174600	NFY-B10 (AT3G53340)			

#### Structure of PvNF-YA genes

The gene structure analysis revealed that members of the NF-YA family have a complex intron/exon organization with 5–7 introns per gene, distributed mainly along the 5′ UTRs and the coding regions, being *PvNF-YA6* the only member with an intron in the 3′ UTR region (Figure 1A). Notably, all *PvNF-YA* members contain at least 1 intron in the 5′ UTR region. A previous report has shown that the *NF-YA1* gene of *M. truncatula* (formerly *MtHAP2-1*) is subjected to a post-transcriptional regulatory mechanism that involves a short peptide that destabilizes the mRNA. This peptide is encoded by an upstream open reading frame (uORF) produced by an alternative spliced variant of *NF-YA1* that retains a long intron in the 5′ UTR region (Combier et al., 2008). The presence of introns in the 5′ UTR regions of all common bean *NF-YA* members prompted us to investigate whether uORFs are encoded within the sequence upstream of the main ORF when introns are retained. We identified uORFs (≥60 nt) in 5 out of 9 *NF-YA* genes. *PvNF-YA1* and *-YA2* contain 2 uORFs, *PvNF-YA4* contains 4 uORFs, and *PvNF-YA8* and *-YA9* contain only 1 uORF when introns present at the 5′ UTR are retained (Figure S2). *NF-YA* transcripts are also regulated at postranscriptional level by the action of microRNA169 (miR169), which binds to the 3′ UTR region of the NF-YA transcripts and promotes their cleavage by the slicing protein Argonaute 1 (Jones-Rhoades and Bartel, 2004; Combier et al., 2006; Li et al., 2008). This regulatory mechanism is conserved among angiosperms, including the *Leguminosae* family (Cuperus et al., 2011). A recent report identified 7 isoforms of the miR169 family in common bean, being miR169b, miR169c and miR169d the most abundant 21 nt isoforms (Pelaez et al., 2012). Therefore, we investigated whether the 9 annotated members of the *PvNF-YA* family (Table 1) contained putative target sites for miR169b/c/d. For this analysis we used the Target-align algorithm (Xie and Zhang, 2010), allowing up to four mismatches in the mRNA:miRNA duplex. With the exception of *PvNF-YA2, -YA6*, and *-YA8*, all members of the *PvNF-YA* family contain at least one target site for miR169b, miR169c, and/or miR169d in their 3′ UTRs (Figure S3). As previously observed in *NF-YA1* of *M. truncatula* (Combier et al., 2006), the 3′ UTR of *PvNF-YA1* exhibits the so called “two hit” model of dual miRNA target sites for either miR169b or miR169d.

#### Structure of PvNF-YB and PvNF-YC genes

In contrast to the *PvNF-YA* family, genes of the *PvNF-YB* family were less structured, showing a variable intron/exon organization among the different members of this family (Figure 1B). Introns were detected only in 5 out of the 14 genes of this family, being *PvNF-YB2* and *-YB5* the most structured ones with 6 introns each, followed by *PvNF-YB14* with 4 and *PvNF-YB10* and *-YB11* with only 1 intron each. These introns were distributed along the 5′ UTR and the coding region of these genes. On the other hand, the analysis of the NF-YC family revealed that 4 (*PvNF-YC1,-YC2*, *-YC4*, or *-YC6*) out of the 7 *PvNF-YC* members contain at least one intron. *PvNF-YC1* contains 3 introns, 2 of them located in the 5′ UTR region and the third one located in the 3′ UTR. In the case of *PvNF-YC2* and *-YC6*, introns are detected exclusively in the 5′ UTR region, whereas the only intron detected in *PvNF-YC4* is located in the coding region (Figure 1C). For *PvNF-YC7*, 5′ or 3′ UTR regions were not defined in the gene models proposed in the current version of the common bean genome or in the EST database.

### Multiple alignment and phylogenetic analysis of PvNF-Y families

**Figure 2:**
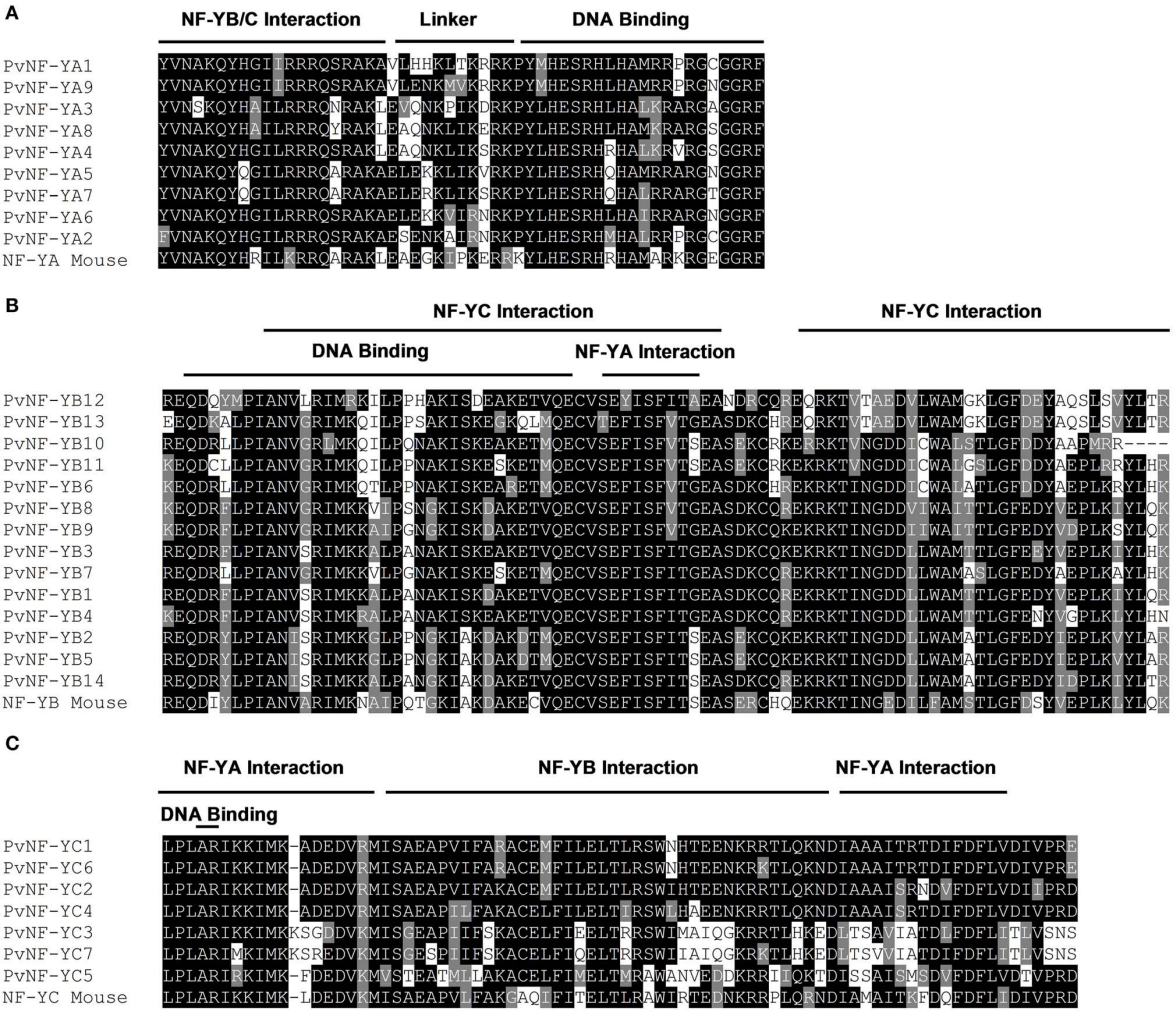
**Multiple alignments of conserved regions of PvNF-Y families**. Sequence alignments among the highly conserved domains of NF-YA **(A)**, NF-YB **(B)**, and NF-YC **(C)** proteins of common bean (Pv) and *Mus musculus* (Mouse). Multiple protein sequence alignments were generated by ClustalW (Thompson et al., 1994). Conserved regions were shading using BOXSHADE 3.21 to produce a graphic representation of the alignment. Identical amino acids are highlighted in black boxes, and similar residues are highlighted in gray boxes. The DNA-binding domain and the domains required for the interaction with the other subunits previously defined in yeast and mammals are indicated.

**Figure 3:**
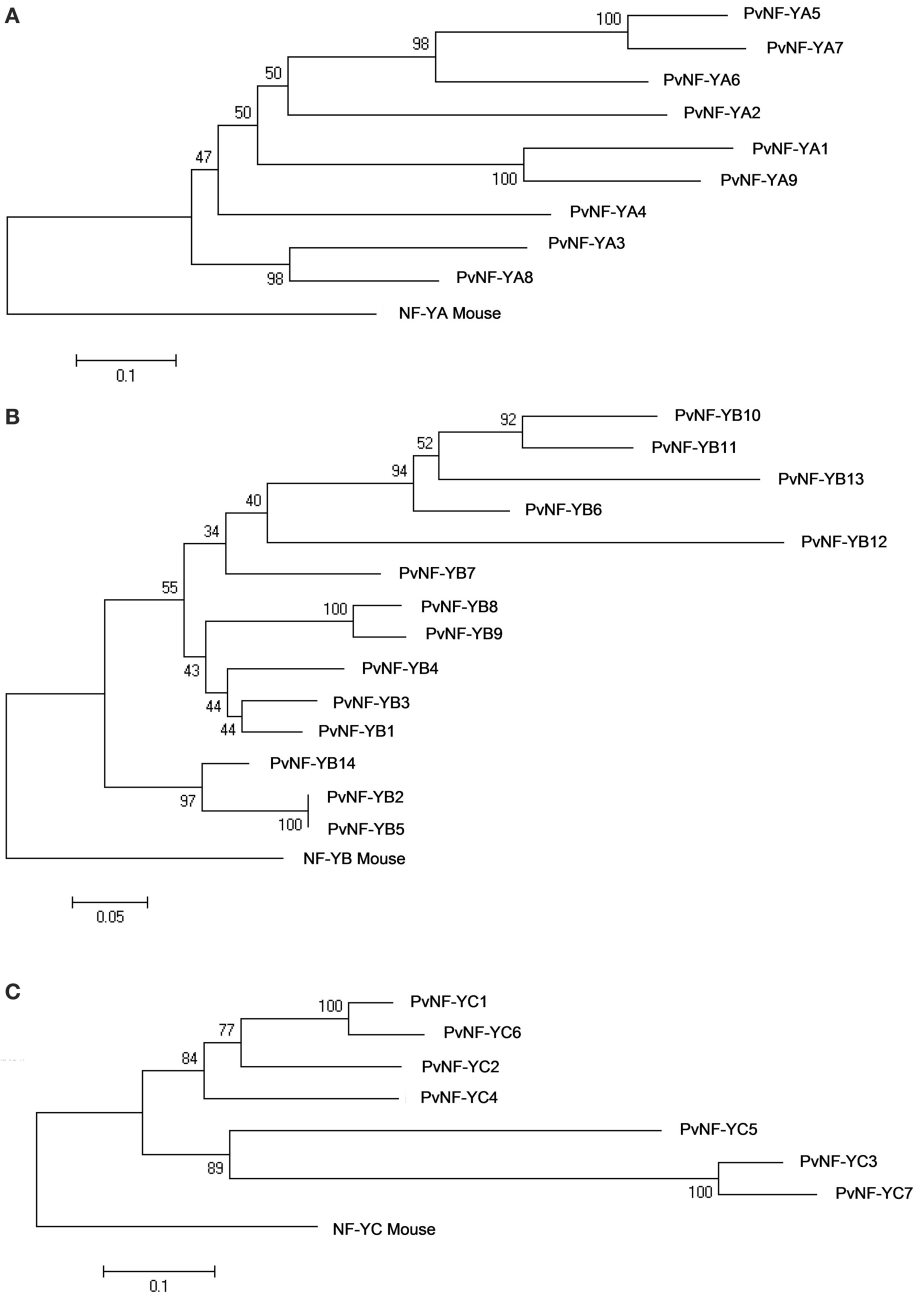
**Phylogenic trees for the PvNF-Y families**. Phylogenetic trees for the NF-YA **(A)**, NF-YB **(B)**, and NF-YC **(C)** families were constructed using the neighbor-joining method based on the multiple sequence alignment analysis of the full-length proteins obtained from the common bean genome V1.0 database, including the mouse NF-Y amino acid sequences as root (rooted tree). All trees were determined and constructed using MEGA 5 from a ClustalW analysis. Reliability values at each branch represent bootstrap samples (10,000 replicates).

Multiple alignments for each PvNF-Y subunit families were constructed using ClustalW (Thompson et al., 1994). As previously described in other plant species, PvNF-Y proteins of each family show a central core region of extensive homology flanked by non-conserved sequences (Figures S4–S6). Based on studies performed in yeast and mammals, this central region contains the conserved domains required for subunit interactions and DNA binding (Xing et al., 1993, 1994; Kim et al., 1996; Sinha et al., 1996; Mcnabb et al., 1997). Alignments of the central core regions are depicted in Figure 2. The multiple alignments of entire protein sequences generated for each PvNF-Y family were used to developed neighbor-joining phylogenetic trees (Figure 3). In addition, we developed phylogenetic trees for each NF-Y family comparing proteins of *Arabidopsis*, *M. truncatula*, soybean and common bean (Figure S7).

#### PvNF-YA family

PvNF-YA proteins are variable in length, ranging between 234 and 349 amino acids. The conserved central region is composed of 53 amino acids and contains two characteristic domains with separated functions (Figure 2A). Studies carried out in metazoans and yeast indicated that the first domain is required for subunit interactions, whereas the second one is important for DNA binding and sequence recognition specificity (Xing et al., 1993, 1994; Mantovani et al., 1994). Outside this conserved central region, limited amino acid conservation was observed among different members of this family. Previous studies have shown that regions flanking the conserved central domain of NF-YA proteins are characterized by the presence of Gln (Q) and Ser/Thr (S/T) rich- regions, which presumably act in transcriptional activation (Coustry et al., 1996; De Silvio et al., 1999). As in other eukaryotes, the alignment presented in Figure S4 indicates that regions flanking the conserved domain of PvNF-YA proteins contain a high composition of Q and S/T residues; however, the number and position of these residues are variable between the different members of this family. The phylogenetic tree of the PvNF-YA family using full length proteins identified two main clades, one containing only 2 members and another one containing 7 members (Figure 3A). The identity matrix of full length PvNF-YA proteins generated by ClustalW (Table S3) indicated that some pairs of proteins exhibit higher amino acid identity with each other (e.g., PvNF-YA5 and -YA7, PvNF-YA1 and -YA9, or PvNF-YA3 and -YA8 exhibit 71, 63, or 61% of identity, respectively) than with the rest of the members in this family. The microsynteny analysis supported these phylogenetic relationships among PvNF-YA duplicates (Figure S8A). The ratio of non-synonymous (Ka) and synonymous (Ks) substitution rates estimated was less than 1 for the majority of the PvNF-YA duplicates found in syntenic regions, suggesting that these genes were subjected to purifying or negative selection (e.g., most amino acid changes are deleterious and, therefore, are selected against).

#### PvNF-YB family

The PvNF-YB family is composed of 14 members of variable length (132–229 amino acids), which are highly similar in the 96 amino acids that define the conserved central domain involved in the interaction with NF-YC and NF-YA subunits and DNA binding (Figure 2B). This conserved domain exhibits structural and sequence similarity to the HFD of histone H2B. Outside the central region, the sequences of the different PvNF-YB members are variable in length and amino acid composition (Figure S5). The phylogenetic tree of the common bean NF-YB family is divided into 2 main clades (Figure 3B). One of these clades contains only 3 members, 2 of which are identical in amino acid composition (PvNF-YB2 and -YB5). The second clade contains 11 members, with only few of them showing high sequence similarity at amino acid level among each other, e.g., PvNF-YB8 and -YB9 or PvNF-YB10 and -YB11 with 77 and 80% of identity, respectively (Table S4). The microsynteny results illustrated in Figure S8B indicated that PvNF-YB8 and -YB9, as well as PvNF-YB10 and -YB11 loci, are contained in large syntenic regions (*E*-values 3e^−150^ and 0.0, respectively), supporting the phylogenetic relationships among these members. Previous studies in other plant species have showed that some NF-YB members are closely related to the metazoan counterpart, whereas other are more divergent, suggesting that this family might have experienced relaxed selective constrains that resulted in an asymmetric evolution of NF-YB duplicates in plants (Yang et al., 2005; Cao et al., 2011). The results presented in Figure 3B and Figure S7B indicated that this might be also the case in common bean.

#### PvNF-YC family

The PvNF-YC family contains a central domain of 80 amino acids that is highly conserved across the different members, but also with the NF-YC mouse counterpart (Figure 2C). This central domain contains an HFD closed related to histone H2A. The use of mutants in yeast and mammals confirmed the importance of this domain in both DNA binding and NF-Y subunit interactions (Romier et al., 2003). As previously observed in other plant species (Siefers et al., 2009; Cao et al., 2011; Petroni et al., 2012), both the N- and C-terminal domains of most PvNF-YC proteins are characterized by an overall enrichment in Q residues, being PvNF-YC3 and -YC7 the exceptions (Figure S6). Functions in transcriptional activation have been assigned to the Q rich regions of mammal and yeast NF-YC proteins (Coustry et al., 1996; De Silvio et al., 1999). The phylogenetic tree presented in Figure 3C revealed 2 different clades. One of these clades includes 3 members, being PvNF-YC3 and -YC7 highly similar to each other (87% of identity); whereas the second clade contains 4 members, where PvNF-YC1 and -YC6 are more closely related, with 86% of identity (Table S5). The microsynteny analysis of this family indicated that PvNF-YC1 and -YC6 loci are contained in a large syntenic region, supporting the close phylogenetic relationship between these PvNF-YC duplicates (Figure S8C). The estimated Ka/Ks ratios for these PvNF-Y duplicates was 0.13, suggesting that these genes were also subjected to purifying selection.

### Expression analysis of PvNF-Ys in different organs and during the symbiotic association with *R. etli*

Individual members of the NF-Y subunits have shown a tissue specific expression pattern in other plant species (Stephenson et al., 2007; Thirumurugan et al., 2008; Siefers et al., 2009; Cao et al., 2011; Liang et al., 2014). Therefore, we aimed to characterize the expression of different members of each NF-Y subunit in both photosynthetic and non-photosynthetic organs of common bean plants by reverse transcription followed by quantitative PCR (RT-qPCR) using gene specific primers (Table S1). In addition, we aimed to identify *PvNF-Y* genes that are differentially expressed in a more efficient interaction with rhizobia as compared with a less efficient one.

#### PvNF-YA expression

**Figure 4:**
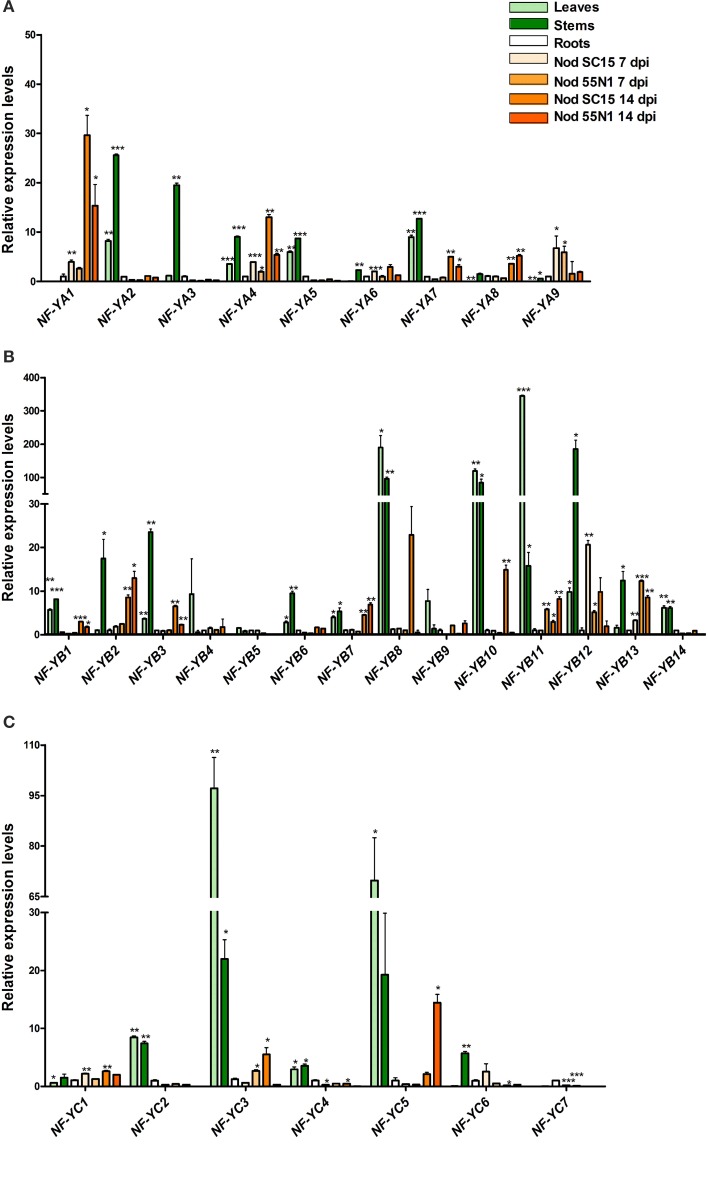
**Expression analysis of NF-Y family members in different organs**. Relative transcript levels obtained by reverse-transcriptase quantitative polymerase chain reaction (RT-qPCR) of *PvNF-YA* **(A)**, *PvNF-YB* **(B)**, and *PvNF-YC* **(C)** gene family members in different organs: leaves, stems, roots, and nodules of 7 or 14 days post-inoculation (dpi) with *Rhizobium etli* SC15 (Nod SC15 7dpi) or 55N1 (Nod 55N1 7dpi). Expression levels were normalized to elongation factor 1α (eEF1α) values and are presented as relative to the root sample. Error bars represent standard deviation (SD) of at least three technical replicates. Results are representative of two independent biological experiments. Single, double, and triple asterisks indicate that values are significantly different from the control value in an unpaired two-tailed *t*-test with *p* < 0.05, 0.01, and 0.001, respectively. Note that in **(B,C)** the Y axis was segmented to accommodate the expression values of genes that are expressed at much higher levels n shoot than in root tissue.

**Figure 5:**
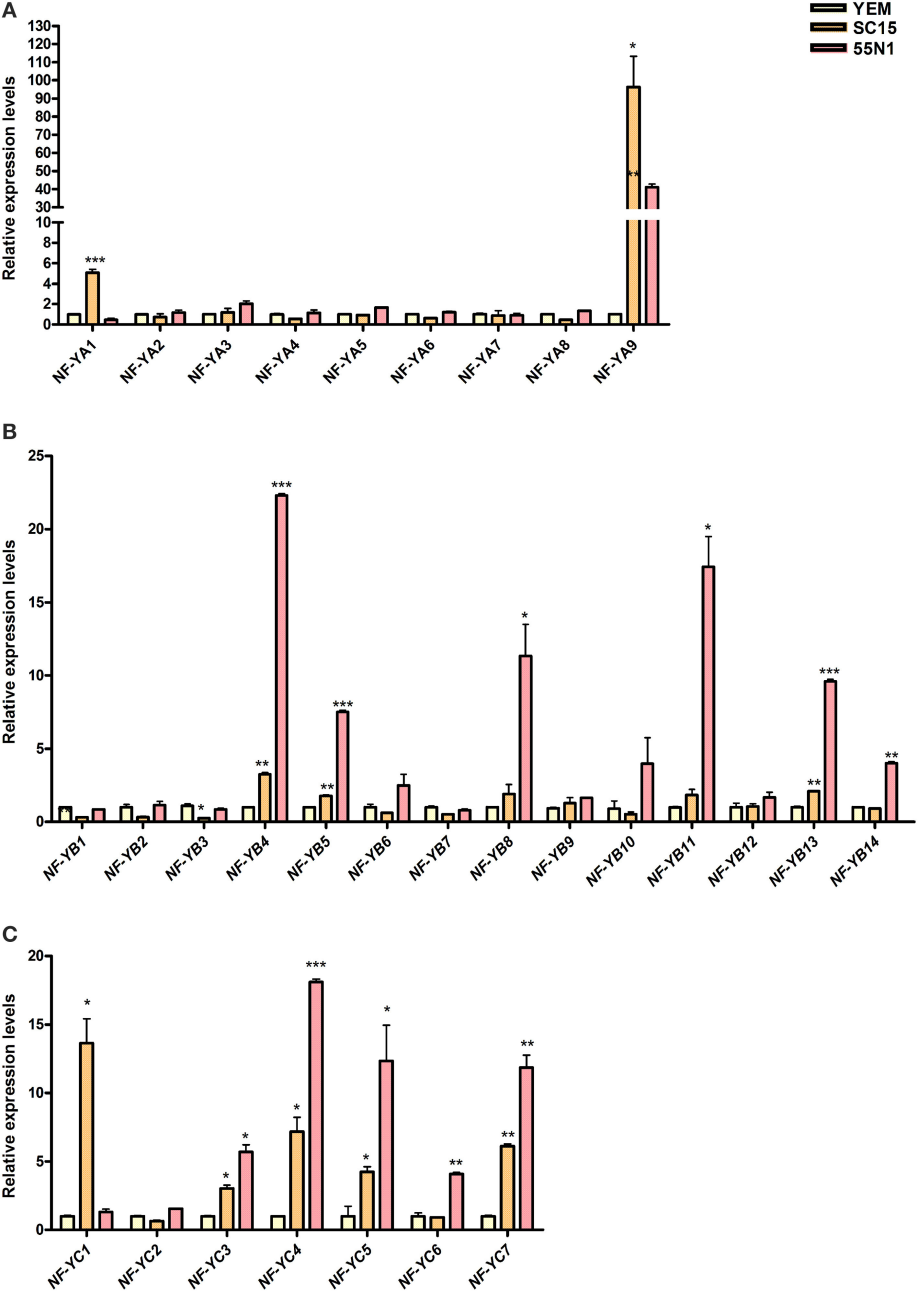
**Expression analysis of PvNF-Y family members in common bean roots upon inoculation with *R. etli* strains SC15 or 55N1**. Relative transcript levels of *PvNF-YA* **(A)**, *PvNF-YB*, **(B)**, and *PvNF-YC* **(C)** in roots 24 h post-inoculation with either strain SC15 or 55N1 of *R. etli* or with the medium used to growth the bacteria (YEM) as control, as determined by RT-qPCR. Expression data were normalized to eEF1α and presented as relative to roots inoculated with YEM. Error bars represent SD of at least three technical replicates. Results are representative of two independent biological experiments. Single, double, and triple asterisks indicate that values are significantly different from the control value in an unpaired two-tailed *t*-test with *p* < 0.05, 0.01, and 0.001, respectively.

Analysis of the PvNF-YA family revealed that all members have detectable transcript levels in at least one of the organs examined (Figure 4A). *PvNF-YA2*, *-YA4*, *-YA5*, and *-YA7* mRNAs accumulated at higher levels in leaves (>6-fold) and stems (>9-fold) as compared to roots. On the other hand, transcript levels of *PvNF-YA3* were 20 fold higher in stems than in leaves or roots. *PvNF-YA6*, *-YA8*, and *-YA9* transcripts were detected in stems and roots, but barely detected in leaf tissues. Notably, transcript accumulation of *PvNF-YA1* was observed in roots, but not in photosynthetic tissues. On the other hand, nodules of 14 dpi with *R. etli* SC15 accumulated significantly higher mRNAs levels of *PvNF-YA1*, *-YA4*, *-YA7*, and *-YA8* as compared to roots or nodules of 7 dpi, whereas *PvNF-YA9* mRNA levels were higher in nodules of 7 dpi than in roots or older nodules. Interestingly, expression of both *PvNF-YA1* and *-YA4* was lower in nodules formed with the less efficient strain 55N1, whereas no significant differences in the expression of *Pv-NF-Y9* were observed between nodules occupied by SC15 or 55N1. In order to identify genes that are regulated during early times of the symbiotic interaction, we analyzed gene expression in roots at 24 hpi with *R. etli*. *PvNF-YA1* and *-YA9* significantly increased in response to rhizobia, whereas the rest of the members did not significantly change in the tested conditions (Figure 5A). *PvNF-YA1* mRNA levels increased about five-fold upon inoculation with the high efficient strain SC15, but did not significantly change with 55N1. On the other hand, *PvNF-YA9* increased with both strains, although the magnitude of the increase was higher with SC15 than with 55N1 (>90- vs. 40-fold).

#### PvNF-YB expression

Analysis of the *PvNF-YB* family revealed that seven members, *PvNF-YB1*, *-YB6*, *-YB8*, *-YB10*, *-YB11*, *-YB12*, and *-YB14*, were expressed at higher levels in photosynthetic tissues as compared with below-ground plant organs; i.e., roots and nodules (Figure 4B). On the other hand, *PvNF-YB2* and *-YB3* exhibited higher levels of transcripts in stems than in leaves, roots and nodules, whereas expression of *PvNF-YB1*, *-YB2*, -*YB7* was higher in nodules of 14 dpi than in roots or nodules of 7 dpi. Expression of *PvNF-YB10*, *-YB12*, and *-YB13* was very low in roots, but higher in nodules of 14 dpi formed by the strain SC15 as compared to those formed by the strain 55N1. In particular, *PvNF-YB12* transcript levels were also higher in young nodules (7 dpi) formed by SC15 than by 55N1. The expression analysis at an early time point of the symbiotic interaction identified several NF-YB members that significantly increased their transcript levels in response to rhizobia. Notably, most of these transcripts increased to higher levels (e.g., *PvNF-YB4*, *-YB5*, and *-YB13*) or exclusively (e.g., *PvNF-YB8*, *-YB11*, and *-YB14*) with the strain 55N1 as compared to strain SC15, at least at the time point analyzed here (Figure 5B). On the other hand, two members of this family, *PvNF-YB1* and *-YB3*, significantly decreased their levels upon inoculation with strain SC15.

#### PvNF-YC expression

The expression analysis of PvNF-YC members in different organs (Figure 4C) revealed that *PvNF-YC1* is expressed in all selected tissues, in agreement with that previously reported (Peltzer Meschini et al., 2008). Transcripts of three members (*PvNF-YC2*, *-YC3*, and *-YC5*) of this family accumulated at much higher levels (from 10- to 100-fold) in leaf and stem tissues than in roots or nodules. On the other hand, *PvNF-YC4* transcript levels were moderately higher in leaves and stems as compared to roots, and decreased in nodules of 7 and 14 dpi. *PvNF-YC6* transcripts were almost undetectable in leaves, but showed high levels in stems, whereas *PvNF-YC7* mRNAs were detected at higher levels in roots than in nodules or photosynthetic tissues. The analysis of the PvNF-YC family at early stages of the symbiotic interaction revealed that *PvNF-YC1* increased exclusively in response to strain SC15 as previously described (Peltzer Meschini et al., 2008; Zanetti et al., 2010), whereas *PvNF-YC6* was induced only in roots inoculated with strain 55N1 (Figure 5C). On the other hand, four *PvNF-YC* members (*PvNF-YC3*, *Y-C4*, *Y-C5*, and *Y-C7*) presented augmented transcript levels in response to both strains; although, as observed for some *PvNF-YB* members, the fold change in gene expression was higher in roots inoculated with strain 55N1 than in those inoculated with SC15.

## Discussion

In the past decade, plant NF-Y genes have gained major interest due to their roles in plant development or in the response to changing environmental conditions, particularly in the root nodule symbiosis. In this work, we have annotated and characterized 9, 14 and 7 members of *PvNF-YA*, *PvNF-YB*, and *PvNF-YC* families, respectively (Table 1). The number of genes identified for each family in common bean is similar to that of rice, but lower than that previously reported in Arabidopsis and in the model legume *M. truncatula* (Laloum et al., 2014). This might be explained by a contraction of these gene families in common bean, as well as by the incompleteness of the common bean genome sequence (Schmutz et al., 2014).

The gene-structure analysis of the *PvNF-YA* gene family revealed that all members of this family contain five or more introns distributed in their 5′, 3′ UTR or within the coding region (Figure 1). Consistently, it has been recently reported that *NF-YA* genes of *Arabidopsis* and canola are also highly structured (Liang et al., 2014). Five members of the *PvNF-YA* family contain uORFs in their 5′ UTR when introns are retained (Figure S2). These uORFs might either act limiting the translation of the main ORFs (Juntawong et al., 2014) or lead to the synthesis of putative small peptides that destabilize *NF-YA* transcripts (Combier et al., 2008). In *M. truncatula*, *Arabidopsis* and rice, most members of the *NF-YA* family have at least 1 intron in the 5′ UTR region (Laloum et al., 2013), suggesting that this might be a conserved regulatory mechanisms among plant *NF-YA* genes. It has been described that some uORFs encode peptides that are conserved among flowering plants (CPuORFs), which might control translation of downstream ORF in response to small molecules (Hayden and Jorgensen, 2007; Jorgensen and Dorantes-Acosta, 2012). Transcription factors represent approximately 30% of the genes that possess CPuORFs. However, we found that the peptide encoded by the uORF1 of *M. truncatula NF-YA1* gene (Combier et al., 2008) is non-conserved in other legumes (e.g., common bean and soybean) and non-legume species (e.g., *Arabidopsis* and rice), since a TBLASTN search using the *M. truncatula* uORF1 predicted amino acid sequence against the genome databases of these other species did not retrieved any hit. The analysis of the intron/exon organization of *PvNF-YB* and *PvNF-YC* family members indicated that most of these genes are less structured than NF-YA members (Figure 1). This result is in accordance to that observed in *Arabidopsis* and canola. On the contrary, rice NF-YAs, NF-YBs and NF-YCs have different gene structures from *Arabidopsis*, canola and common bean (Liang et al., 2014).

*NF-Y* genes showed high variability in their expression patterns (Figures 4, 5). Some of them showed an ubiquitous expression (e.g., *NF-YA4* and *NF-YC1*), whereas others exhibited an organ specific expression pattern (e.g., *NF-YA1* and *NF-YA9* in roots and nodules, *NF-YA2*, *NF-YB14*, and *NF-YC2* in photosynthetic tissues). Based on the phylogenetic trees presented in Figure S7, some of these NF-Y genes expressed at higher levels in leaves or stems as compared to root tissues (e.g., *PvNF-YB1*, *-YB3*, *-YB4*, *-YB8*, *-YC2*, and *-YC5*) have high sequence similarity with Arabidopsis members implicated in the response to the endoplasmic reticulum stress and/or in the promotion of flowering, such as *AtNF-YB3*, *AtNF-YB2* and *AtNF-YC2* (Petroni et al., 2012; Laloum et al., 2013).

In the context of the root nodule symbiosis, we identified two members of the *PvNF-YA* family, *PvNF-YA1* and *-YA9*, which are highly expressed in nodules as compared to other tissues (Figure 4A). Both *PvNF-YA1* and *-YA9* are differentially induced by the high efficient strain at an early stage of the interaction (Figure 5A), suggesting that these genes might play a role in partner selection, as previously observed for *PvNF-YC1* (Zanetti et al., 2010). PvNF-YA1 and PvNF-YA9 proteins are closely related to the products of *MtNF-YA1* and *MtNF-YA2* genes, respectively, and to the product of the *LjNF-YA1* gene (see Figure S7 and Soyano et al., 2013), which are required for the development of indeterminate and determinate types of nodules in *M. truncatula* and *L. japonicus*, respectively (Combier et al., 2006, 2008; Soyano et al., 2013). In addition, *MtNF-YA1* and *MtNF-YA2* are required for bacterial infection and for the induction of early nodulation genes, such as *ERN1* and *ENOD11* (Laloum et al., 2014; Laporte et al., 2014). However, down regulation of *NF-YA1* did not alter the number of infection events in *L. japonicus* roots. These evidences indicated that *NF-YA1* genes might have evolved to play slightly different functions in these two legumes. Thus, it will be of interest to elucidate the function of *PvNF-YA1* and *Pv-NF-YA9* at different stages of the symbiotic association between common bean and *R. etli*. Similarly, we identified three members of the PvNF-YB family, *PvNF-YB8*, *-YB10*, and *-YB12*, whose transcripts exhibited high levels in nodules formed by strain SC15 as compared to roots, although they are not up-regulated by SC15 at an early stage of the interaction (Figures 4B, 5B). These *PvNF-YB* genes are likely candidates to play a role in nodule development at late stages of the highly efficient association between *P. vulgaris* and the *R. etli* strain SC15. Interestingly, a phylogenetic analysis reported by Soyano et al. (2013) indicated that PvNF-YB10 protein is in the same clade that LjNF-YB1, which physically interacts and functions with LjNF-YA1 in the promotion of root cell divisions and the transcriptional activation of the cell cycle gene *cyclin B*. Previously, we have shown that *PvNF-YC1* is required for the up-regulation of G2/M transition cell cycle genes- including *cyclin B*- in response to rhizobia, and the overexpression of *PvNF-YC1* increased nodule number in *P. vulgaris* roots (Zanetti et al., 2010). Our analysis failed to identify *PvNF-YB* family members that are exclusively or extensively induced by strain SC15 at 24 hpi; however, we can not exclude that some members might be differentially regulated by this strain at other time points, since *PvNF-YB8, -YB10*, and *-YB12* mRNAs accumulated at higher levels in nodules formed by SC15 than in those formed by 55N1 (Figure 4B). Future studies focused on subunit interactions will help to discover whether PvNF-YC1, PvNF-YA1, or -YA9 and PvNF-YB8, -YB10, or YB-12 function in the same heterotrimeric complex to control cell cycle genes and promote the cortical cell divisions that lead to nodule primordia formation.

## Conclusions

In the past 10 years, an increasing amount of genetic and biochemical evidences have supported the importance of the NF-Y family of proteins in different developmental processes along the lifespan of plants, as well as in their adaptation to adverse environmental conditions. Here, we have generated an initial dataset of sequences, intron/exon arrangements and expression patterns of genes encoding NF-Y subunits in *P. vulgaris*, the most important grain legume used for direct human consumption. The phylogenetic relationships and expression patterns generated here constitute a starting point to elucidate the function of the different members of the NF-Y family in morphogenetic programs in *P. vulgaris*, such as the epidermal infection events and nodule organogenesis, as well as to understand how Mesoamerican plants discriminate and select the most efficient rhizobial strains. In addition, the identification of NF-Y subunits that are co-expressed in the same organ or at the same stage of the symbiotic interaction will facilitate the analysis of putative subunit combinations that form functional NF-Y complexes in this legume.

### Conflict of interest statement

The authors declare that the research was conducted in the absence of any commercial or financial relationships that could be construed as a potential conflict of interest.

## Supplementary material

Figure S1 **Chromosomal locations of PvNF-Ys**. The chromosomal position of each NF-YA (red), NF-YB (green) and NF-YC (blue) family members was mapped to the common bean genome using the Map-View function of PGDD. The chromosome number is indicated at the top of each chromosome. The scale is 2 Mb.

Figure S2 **Presence of uORFs in the *PvNF-YA* family members**. Representation of upstream and main open reading frames (uORFs and mORFs, respectively) of individual members of PvNF-YA subunit. The analysis was performed with the VectorNTI Advance 10 program using default parameters and a minimum ORF size ≥60 nucleotides (nt). Lines represent introns, gray boxes represent 5′ UTRs, black arrows represent the mORFs and blue arrows represent uORFs present when intron are retained. Sizes of intron, exons, and ORFs can be estimated using the scale bar (200 bp).

Figure S3 **miR169 target sites in the 3′ UTR of *PvNF-YA* transcripts**. Putative target sites of miR169b/c/d within the 3′ UTR of *PvNF-YA* transcripts were predicted with Target-align (Xie and Zhang, 2010) using default parameters and a maximum of 4 mismatches. The target sites of *PvNF-YA* transcripts are shown at the top and the miRNA at the bottom. Sequence location of the transcript is indicated in gray over the sequence. Perfectly complementary bases are shown in black and connected with solid lines, G:U wobble are indicated in blue and connected with doted lines. Mismatches are shown in pink.

Figure S4 **Multiple alignments of entire protein sequences of PvNF-YA family**. Multiple alignment of the full length NF-YA proteins of common bean and mouse. The analysis was made with the ClustalW program (Thompson et al., 1994) and shaded using BOXSHADE 3.21 to produce a graphic representation of the alignment. Identical amino acids are highlighted in black boxes, and similar residues are highlighted in gray boxes. Numbers on the left indicate the amino acid position on the protein.

Figure S5 **Multiple alignments of entire protein sequences of PvNF-YB family**. Multiple alignment of the full length NF-YB proteins of common bean and mouse. The analysis was performed as described in Figure S3.

Figure S6 **Multiple alignments of entire protein sequences of PvNF-YC family**. Multiple alignment of the full length NF-YC proteins of common bean and mouse. The analysis was performed as described in Figure S3.

Figure S7 **Phylogenetic trees comparing NF-Y families in *P. vulgaris*, *M. truncatula*, *G. max*, and *Arabidopsis***. Phylogenetic relationship of the NF-YA **(A)**, NF-YB **(B)**, and NF-YC **(C)** families of common bean, *M. truncatula, soybean* and *Arabidopsis*. Multiple sequence alignments were generated on full-length proteins with ClustalW (Thompson et al., 1994). Phylogenetic trees were constructed using the neighbor-joining method based on the multiple sequence alignment analysis using MEGA5. Reliability values at each branch represent bootstrap values (10,000 replicates). The mouse NF-Y subunits were used to root the trees. Gene ID codes from the genome database available at TAIR or Phytozome were included. For MtNF-YB18 and MtNF-YC4, which do not have a Gene ID at the *M. truncatula* genome database, GenBank accession numbers were included. The higher number of NF-Y members in soybean as compared to the other plant species can be explained by a recent whole-genome duplication event.

Figure S8 **Colinear blocks of Pv-NF-Y loci in the common bean genome**. Colinear blocks containing PvNF-YA **(A)**, PvNF-YB **(B)**, and PvNF-YC **(C)** loci were obtained using the Locus Search function of PGDD. Structures of colinear blocks are shown. In each case, PvNF-Y loci are indicated by red arrows. Blue arrows are other anchor genes in a display region of ±100 kb. Non-synonymous (Ka) and synonymous (Ks) substitution rates are indicated.
