# Supplementary material for: Annotation, phylogeny and expression analysis of the nuclear factor Y gene families in common bean (Phaseolus vulgaris)
Source: Front Plant Sci. 2015 Jan 14;5:761. doi: 10.3389/fpls.2014.00761 (PMC4294137; doi:10.3389/fpls.2014.00761)
Supplement: Figure S1 — Chromosomal locations of PvNF-Ys. The chromosomal position of each NF-YA (red), NF-YB (green) and NF-YC (blue) family members was mapped to the common bean genome using the Map-View function of PGDD. The chromosome number is indicated at the top of each chromosome. The scale is 2 Mb. [file Presentation1.PDF]

Figure S1

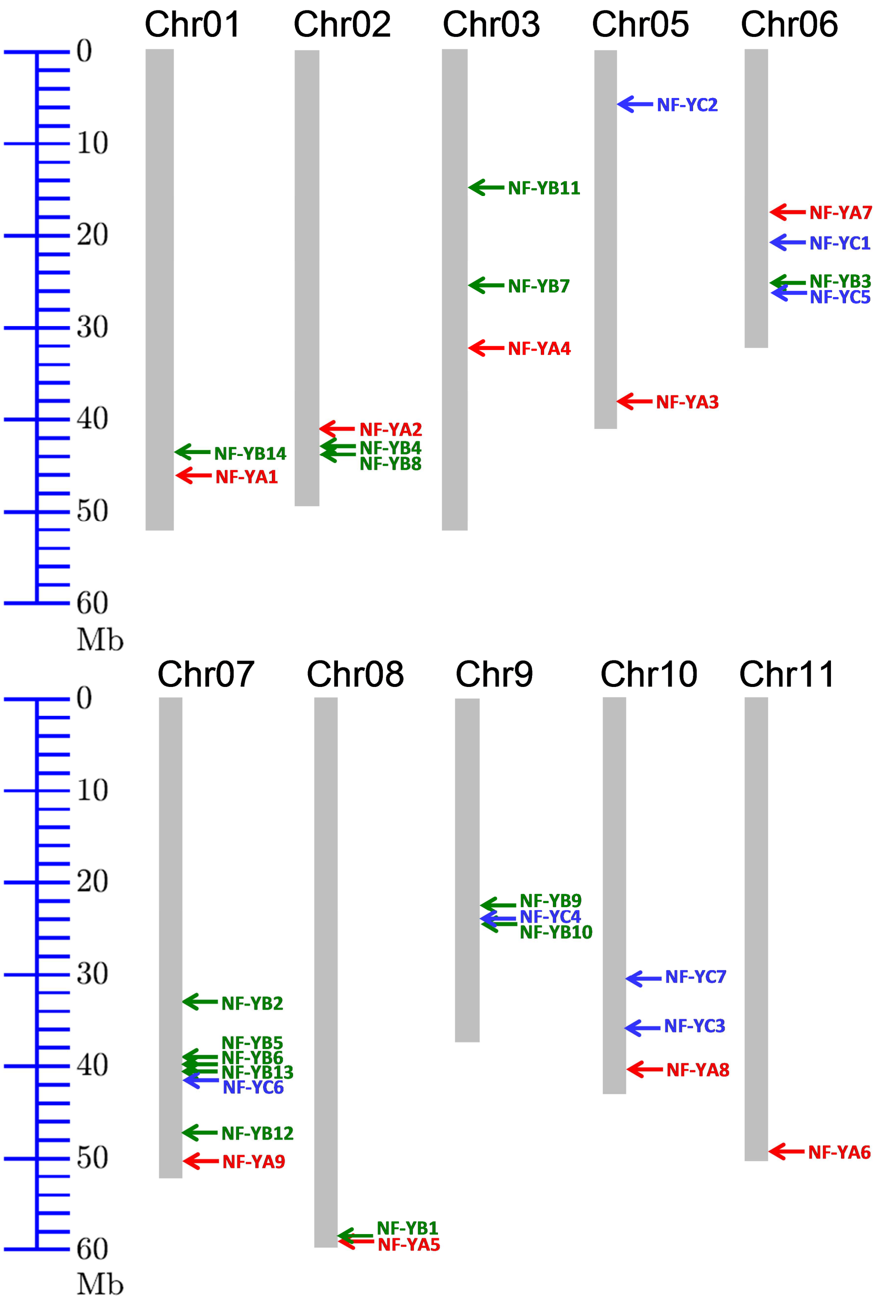
Chr01
Chr02
Chr03
Chr05
Chr06
0
10
20
30
40
50
60
Mb
NF-YC2
NF-YB11
NF-YA7
NF-YC1
NF-YB7
NF-YB3
NF-YC5
NF-YA4
NF-YA3
NF-YA2
NF-YB14
NF-YB4
NF-YB8
NF-YA1
Chr07
Chr08
Chr9
Chr10
Chr11
NF-YB9
NF-YC4
NF-YB10
NF-YC7
NF-YB2
NF-YC3
NF-YB5
NF-YB6
NF-YB13
NF-YC6
NF-YA8
NF-YB12
NF-YA9
NF-YA6
NF-YB1
NF-YA5

Figure S2

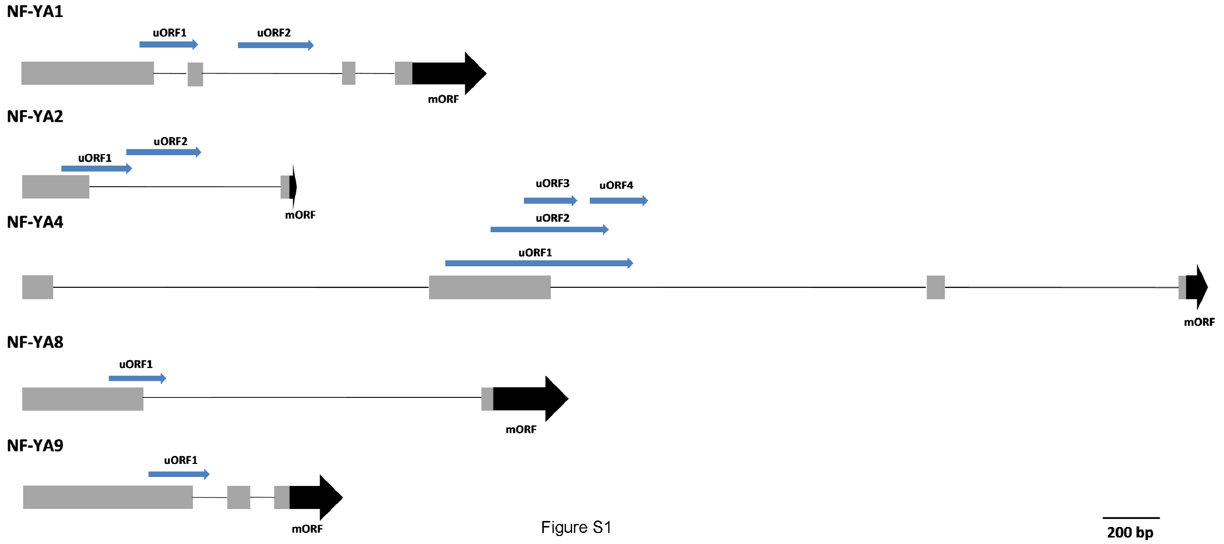
NF-YA1
uORF1
uORF2
mORF
NF-YA2
uORF2
uORF1
mORF
NF-YA4
uORF3
uORF4
uORF2
uORF1
mORF
NF-YA8
uORF1
mORF
NF-YA9
uORF1
mORF
Figure S1
200 bp

Figure S3

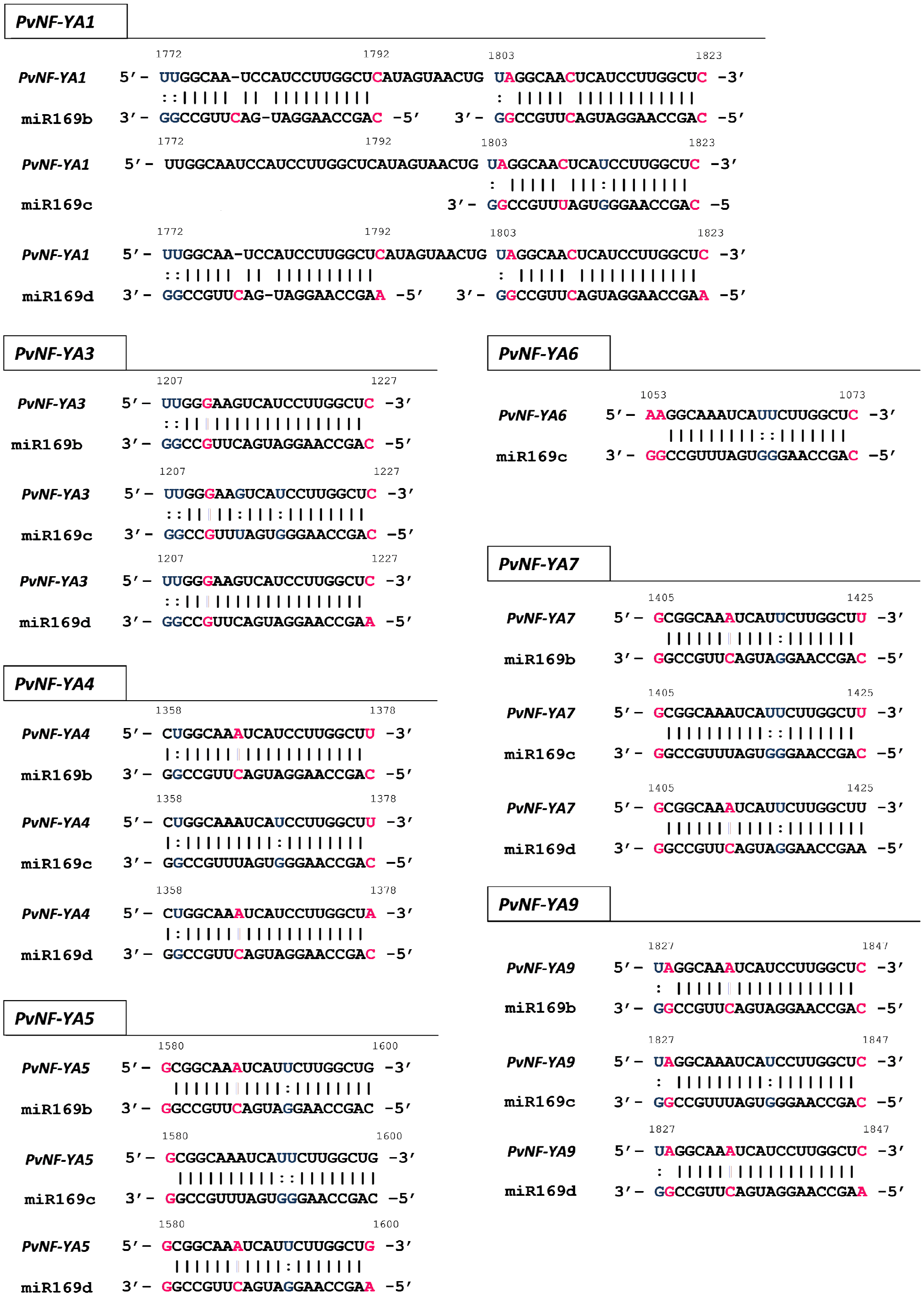
PvNF-YA1
1772 1792 1803 1823
PvNF-YA1 5′- UUGGCAA-UCCAUCCUUGGCUCAUAGUAACUG UAGGCAACUCAUCCUUGGCUC -3′
miR169b 3′- GGCCGUUCAG-UAGGAACCGAC -5′ 3′- GGCCGUUCAGUAGGAACCGAC -5′
PvNF-YA1 5′- UUGGCAAUCCAUCCUUGGCUCAUAGUAACUG UAGGCAACUCAUCCUUGGCUC -3′
miR169c 3′- GGCCGUUUAGUGGGAACCGAC -5
PvNF-YA1 5′- UUGGCAA-UCCAUCCUUGGCUCAUAGUAACUG UAGGCAACUCAUCCUUGGCUC -3′
miR169d 3′- GGCCGUUCAG-UAGGAACCGAA -5′ 3′- GGCCGUUCAGUAGGAACCGAA -5′
PvNF-YA3
1207 1227
PvNF-YA3 5′- UUGGGAAGUCAUCCUUGGCUC -3′
miR169b 3′- GGCCGUUCAGUAGGAACCGAC -5′
PvNF-YA3 5′- UUGGGAAGUCAUCCUUGGCUC -3′
miR169c 3′- GGCCGUUUAGUGGGAACCGAC -5′
PvNF-YA3 5′- UUGGGAAGUCAUCCUUGGCUC -3′
miR169d 3′- GGCCGUUCAGUAGGAACCGAA -5′
PvNF-YA4
1358 1378
PvNF-YA4 5′- CUGGCAAAUCAUCCUUGGCUU -3′
miR169b 3′- GGCCGUUCAGUAGGAACCGAC -5′
PvNF-YA4 5′- CUGGCAAAUCAUCCUUGGCUU -3′
miR169c 3′- GGCCGUUUAGUGGGAACCGAC -5′
PvNF-YA4 5′- CUGGCAAAUCAUCCUUGGCUA -3′
miR169d 3′- GGCCGUUCAGUAGGAACCGAC -5′
PvNF-YA5
1580 1600
PvNF-YA5 5′- GCGGCAAAUCAUUCUUGGCUG -3′
miR169b 3′- GGCCGUUCAGUAGGAACCGAC -5′
PvNF-YA5 5′- GCGGCAAAUCAUUCUUGGCUG -3′
miR169c 3′- GGCCGUUUAGUGGGAACCGAC -5′
PvNF-YA5 5′- GCGGCAAAUCAUUCUUGGCUG -3′
miR169d 3′- GGCCGUUCAGUAGGAACCGAA -5′
PvNF-YA6
1053 1073
PvNF-YA6 5′- AAGGCAAAUCAUUCUUGGCUC -3′
miR169c 3′- GGCCGUUUAGUGGGAACCGAC -5′
PvNF-YA7
1405 1425
PvNF-YA7 5′- GCGGCAAAUCAUUCUUGGCUU -3′
miR169b 3′- GGCCGUUCAGUAGGAACCGAC -5′
PvNF-YA7 5′- GCGGCAAAUCAUUCUUGGCUU -3′
miR169c 3′- GGCCGUUUAGUGGGAACCGAC -5′
PvNF-YA7 5′- GCGGCAAAUCAUUCUUGGCUU -3′
miR169d 3′- GGCCGUUCAGUAGGAACCGAA -5′
PvNF-YA9
1827 1847
PvNF-YA9 5′- UAGGCAAAUCAUCCUUGGCUC -3′
miR169b 3′- GGCCGUUCAGUAGGAACCGAC -5′
PvNF-YA9 5′- UAGGCAAAUCAUCCUUGGCUC -3′
miR169c 3′- GGCCGUUUAGUGGGAACCGAC -5′
PvNF-YA9 5′- UAGGCAAAUCAUCCUUGGCUC -3′
miR169d 3′- GGCCGUUCAGUAGGAACCGAA -5′

Figure S4

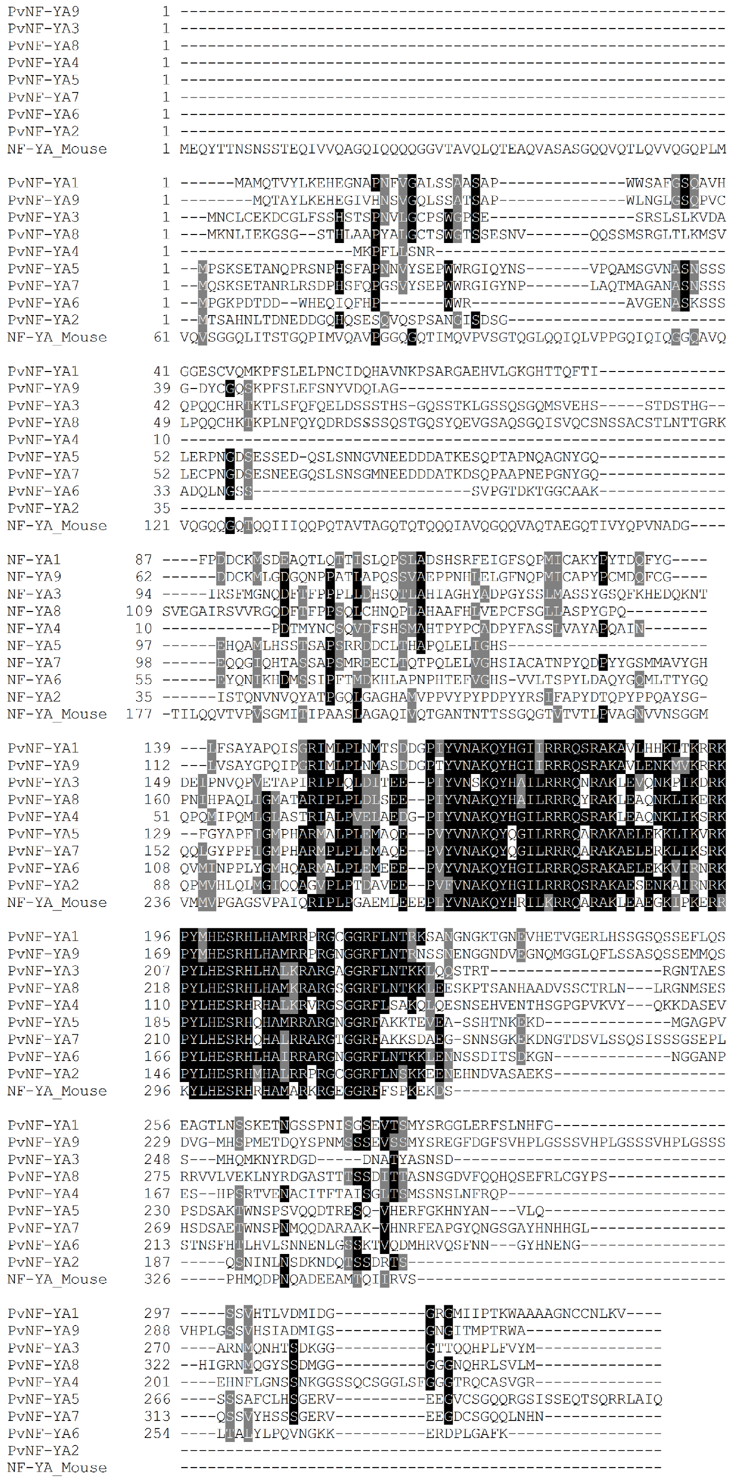

Figure S5

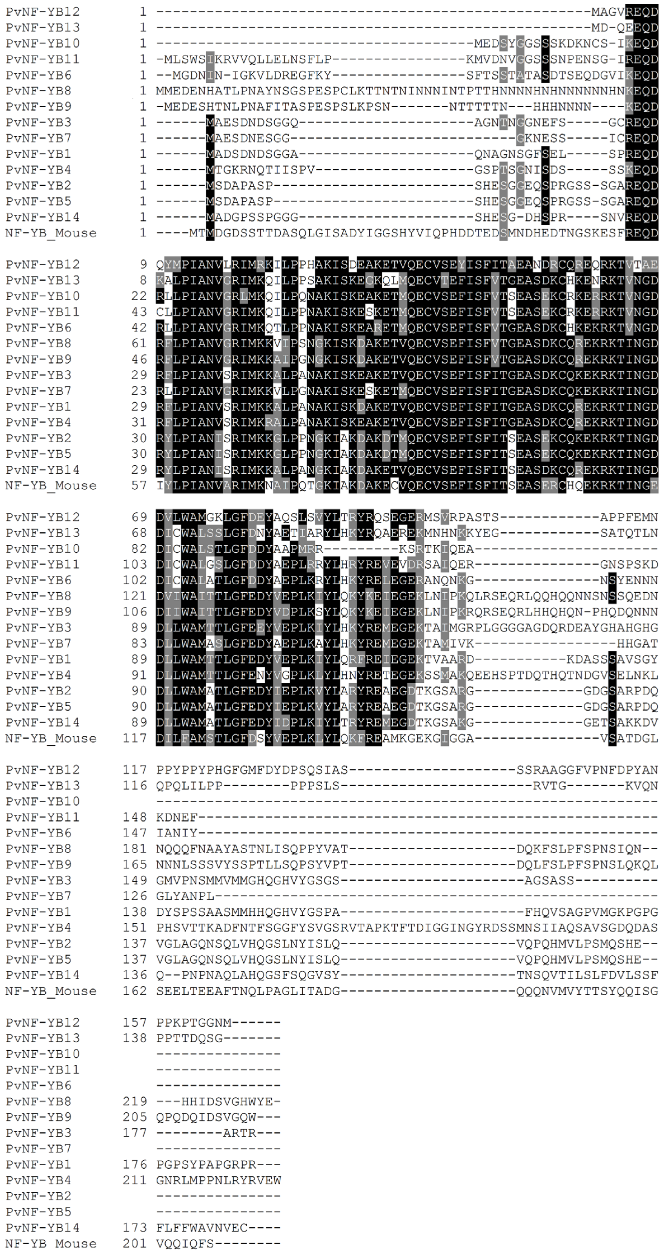
PvNF-YB12 1 --MAGVREQD
PvNF-YB13 1 --MD-QEEQD
PvNF-YB10 1 --------------------------------------MEDSYGGSSSKDKNCS-IKEQD
PvNF-YB11 1 -MLSWSIKRVVQLLELNSFLP----------------KMVDNVGGSSSNPENSG-IREQD
PvNF-YB6 1 --MGDNIN-IGKVLDREGFKY----------------SFTSSTATASDTSEQDGVIKEQD
PvNF-YB8 1 MMEDENHATLPNAYNSGSPESPCLKTTNTNINNNINTPTTHNNNNHNHNNNNNNNHNKEQD
PvNF-YB9 1 -MEDESHTNLPNAFITASPESPSLKPSN-------NTTTTTN---HHHNNNN----KEQD
PvNF-YB3 1 ------MAESDNDSGGQ---------------------AGNTNGGNEFS-----GCREQD
PvNF-YB7 1 ------MAESDNESGG---------------------------GKNESS-----ICREQD
PvNF-YB1 1 ------MADSDNDSGGA---------------------QNAGNSGFSEL-----SPREQD
PvNF-YB4 1 ------MTGKRNQTIISPV-------------------GSPTSGNISDS-----SSKEQD
PvNF-YB2 1 ------MSDAPASP------------------------SHESGGEQSPRGSS-SGAREQD
PvNF-YB5 1 ------MSDAPASP------------------------SHESGGEQSPRGSS-SGAREQD
PvNF-YB14 1 ------MADGPSSPGGG---------------------SHESG-DHSPR----SNVREQD
NF-YB_Mouse 1 ----MTMDGDSSTTDASQLGISADYIGGSHYVIQPHDDTEDSMNDHEDTNGSKESFREQD
PvNF-YB12 9 QYMPIANVLRIMRKILPPHAKISDEAKETVQECVSEYISFITAEANDRCQREQRKTVTAE
PvNF-YB13 8 KALPIANVGRIMKQILPPSAKISKEGKQLMQECVTEFISFVTGEASDKCHKENRKTVNGD
PvNF-YB10 22 RLLPIANVGRLMKQILPQNAKISKEAKETMQECVSEFISFVTSEASEKCRKERRKTVNGD
PvNF-YB11 43 CLLPIANVGRIMKQILPPNAKISKESKETMQECVSEFISFVTSEASEKCRKERRKTVNGD
PvNF-YB6 42 RLLPIANVGRIMKQTLPPNAKISKEARETMQECVSEFISFVTGEASDKCHKEKRKTVNGD
PvNF-YB8 61 RFLPIANVGRIMKKVIPSNGKISKDAKETVQECVSEFISFVTGEASDKCQREKRKTINGD
PvNF-YB9 46 RFLPIANVGRIMKKAIPGNGKISKDAKETVQECVSEFISFVTGEASDKCQREKRKTINGD
PvNF-YB3 29 RFLPIANVSRIMKKALPANAKISKEAKETVQECVSEFISFITGEASDKCQKEKRKTINGD
PvNF-YB7 23 RLLPIANVGRIMKKVLPGNAKISKESKETMQECVSEFISFITGEASDKCQKEKRKTINGD
PvNF-YB1 29 RFLPIANVSRIMKKALPANAKISKDAKETVQECVSEFISFITGEASDKCQREKRKTINGD
PvNF-YB4 31 RFLPIANVSRIMKRALPANAKISKEAKETVQECVSEFISFITGEASDKCQREKRKTINGD
PvNF-YB2 30 RYLPIANISRIMKKGLPPNGKIAKDAKDTMQECVSEFISFITSEASEKCQKEKRKTINGD
PvNF-YB5 30 RYLPIANISRIMKKGLPPNGKIAKDAKDTMQECVSEFISFITSEASEKCQKEKRKTINGD
PvNF-YB14 29 RYLPIANISRIMKKALPANGKIAKDAKETVQECVSEFISFITSEASDKCQREKRKTINGD
NF-YB_Mouse 57 IYLPIANVARIMKNAIPQTGKIAKDAKECVQECVSEFISFITSEASERCHQEKRKTINGE
PvNF-YB12 69 DVLWAMGKLGFDEYAQSLSVYLTRYRQSEGERMSVRPASTS------------APPFEMN
PvNF-YB13 68 DICWALSSLGFDNYAETIARYLHKYRQAEREKMNHNKKYEG------------SATQTLN
PvNF-YB10 82 DICWALSTLGFDDYAAPMRR---------KSRTKIQEA----------------------
PvNF-YB11 103 DICWALGSLGFDDYAEPLRRYLHRYREVEVDRSAIQER---------------GNSPSKD
PvNF-YB6 102 DICWALATLGFDDYAEPLKRYLHKYRELEGERANQNKG---------------NSYENNN
PvNF-YB8 121 DVIWAITTLGFEDYVEPLKIYLQKYKEIEGEKLNIPKQLRSEQRLQQHQQNNSNSSQEDN
PvNF-YB9 106 DIIWAITTLGFEDYVDPLKSYLQKYKEIEGEKLNIPKRQRSEQRLHHQHQN-PHQDQNNN
PvNF-YB3 89 DLLWAMTTLGFEEYVEPLKIYLHKYREMEGEKTAIMGRPLGGGGAGDQRDEAYGHAHGHG
PvNF-YB7 83 DLLWAMASLGFEDYAEPLKAYLHKYREMEGEKTAMIVK-----------------HHGAT
PvNF-YB1 89 DLLWAMTTLGFEDYVEPLKIYLQRFREIEGEKTVAARD-----------KDASSSAVSGY
PvNF-YB4 91 DLLWAMTTLGFENYVGPLKLYLHNYRETEGEKSSMAKQEEHSPTDQTHQTNDGVSELNKL
PvNF-YB2 90 DLLWAMATLGFEDYIEPLKVYLARYREAEGDTKGSARG-------------GDGSARPDQ
PvNF-YB5 90 DLLWAMATLGFEDYIEPLKVYLARYREAEGDTKGSARG-------------GDGSARPDQ
PvNF-YB14 89 DLLWAMATLGFEDYIDPLKIYLTRYREMEGDTKGSAKG-------------GETSAKKDV
NF-YB_Mouse 117 DILFAMSTLGFDSYVEPLKLYLQKFREAMKGEKGIGGA---------------VSATDGL
PvNF-YB12 117 PPYPPYPHGFGMFDYDPSQSIAS--------------------SSRAAGGFVPNFDPYAN
PvNF-YB13 116 QPQLILPP--------PPPSLS-----------------------RVTG-------KVQN
PvNF-YB10 --
PvNF-YB11 148 KDNEF---
PvNF-YB6 147 IANIY---
PvNF-YB8 181 NQQQFNAAYASTNLISQPPYVAT--------------------DQKFSLPFSPNSIQN--
PvNF-YB9 165 NNNLSSSVYSSPTLLSQPSYVPT--------------------DQLFSLPFSPNSLQKQL
PvNF-YB3 149 GMVPNSMMVMMGHQGHVYGSGS----------------------AGSASS----------
PvNF-YB7 126 GLYANPL---
PvNF-YB1 138 DYSPSSAASMMHHQGHVYGSPA----------------------FHQVSAGPVMGKPGPG
PvNF-YB4 151 PHSVTTKADFNTFSGGFYSVGSRVTAPKTFTDIGGINGYRDSSMNSIIAQSAVSGDQDAS
PvNF-YB2 137 VGLAGQNSQLVHQGSLNYISLQ---------------------VQPQHMVLPSMQSHE--
PvNF-YB5 137 VGLAGQNSQLVHQGSLNYISLQ---------------------VQPQHMVLPSMQSHE--
PvNF-YB14 136 Q--PNPNAQLAHQGSFSQGVSY---------------------TNSQVTILSLFDVLSSF
NF-YB_Mouse 162 SEELTEEAFTNQLPAGLITADG---------------------QQQNVMVYTTSYQQISG
PvNF-YB12 157 PPKPTGGNM------
PvNF-YB13 138 PPTTDQSG-------
PvNF-YB10 ---------------
PvNF-YB11 ---------------
PvNF-YB6 ---------------
PvNF-YB8 219 ---HHIDSVGHWYE-
PvNF-YB9 205 QPQDQIDSVGQW---
PvNF-YB3 177 --------ARTR---
PvNF-YB7 ---------------
PvNF-YB1 176 PGPSYPAPGRPR---
PvNF-YB4 211 GNRLMPPNLRYRVEW
PvNF-YB2 ---------------
PvNF-YB5 ---------------
PvNF-YB14 173 FLFFWAVNVEC----
NF-YB_Mouse 201 VQQIQFS--------

Figure S6

```
PvNF-YC1        1 MD------HQGH--GQNPSMGVVGSGAQLTYGSNPYQQGQLTGPPGSVVTSVGTIQSSGQ
PvNF-YC6        1 MD------HQGHGHGQNPSLGVVGSGPQLTYGSNPYQASHITASPGMVVASPGTIQSTGQ
PvNF-YC2        1 MDKSEVTPQRQQQQQQQHVMGVAAGASQMAYS--HYQTAPMVAAGTPAVAVPSQTQAPG-
PvNF-YC4        1 MEN-----------TQQGGQGQSG----------PYAGAAAGSAGAAAGAPP--------
PvNF-YC3        1 -------------------MRQAG----------AYSGLLCGGVSG--RTGP--------
PvNF-YC7        1 -------------------MRQAG----------AYSGLLCGGISG--RTGP--------
PvNF-YC5        1 MDQ-----------NQHGEAGGKGPAPAEEEAPNTNPPTQTVGVTGGNRYDR--------
NF-YC_Mouse     1 -------------MSTEGGFGGTSSSDAQQS-----------------------------

PvNF-YC1       53 PAGAQLGQHQLAY---QHIHQQQQHQLQQQLQQFWSSQYQEIE--KVTDFKNHSLPLARI
PvNF-YC6       55 PAATQLGQHQLAY---QHMHQQQQQQLQQRLQAFWTNQYQEIE--KVTDFKNHSLPLARI
PvNF-YC2       58 --AFSSSAHQLAYQQAQHFHHQQQQHQQQQLQMFWSDQMQEIE--QTIDFKNHSLPLARI
PvNF-YC4       32 ---------------FQHLLHQQ----QQQLQMFWSYQRQEIE--HVNDFKNHQLPLARI
PvNF-YC3       22 ----------------------------------------------------HPLPLARI
PvNF-YC7       22 ----------------------------------------------------HSLPLARI
PvNF-YC5       42 ----------------QCMQQQQQEKMQQRVNSFWARQLKEIE--ESTDLKSHSLPLARI
NF-YC_Mouse    19 ------------------------------LQSFWPRVMEEIRNLTVKDFRVQELPLARI

PvNF-YC1      108 KKIMK-ADEDVRMISAEAPVIFARACEMFILELTLRSWNHTEENKRRTLQKNDIAAAITR
PvNF-YC6      110 KKIMK-ADEDVRMISAEAPVIFARACEMFILELTLRSWNHTEENKRKTLQKNDIAAAITR
PvNF-YC2      114 KKIMK-ADEDVRMISAEAPVIFAKACEMFILELTLRSWIHTEENKRRTLQKNDIAAAISR
PvNF-YC4       71 KKIMK-ADEDVRMISAEAPILFAKACELFILELTIRSWLHAEENKRRTLQKNDIAAAITR
PvNF-YC3       30 KKIMKKSGDDVKMISGEAPIIFSKACELFIEELTRRSWIMAIQGKRRTLHKEDLTSAVIA
PvNF-YC7       30 MKIMKKSREDVKMISGESPIIFSKACELFIQELTRRSWIIAIQGKRKTLHKEDLTSVVIA
PvNF-YC5       84 RKIMK-FDEDVKMVSTEATMLLAKACELFIMELTMRAWANVEDDKRRIIQKTDISSAISM
NF-YC_Mouse    49 KKIMK-LDEDVKMISAEAPVLFAKGAQIFITELTLRAWIRTEDNKRRPLQRNDIAMAITK

PvNF-YC1      167 TDIFDFLVDIVPREDLKDEVLASIPRGTMPVGGPPDALPYCYMQPQHAPQVGTAGVIMG-
PvNF-YC6      169 TDIFDFLVDIVPREDYKDEVLASMPRGTVPVTGAPEALPYCYMPPQHA-QVGTAAVMMGN
PvNF-YC2      173 NDVFDFLVDIIPRDELKEEGLG-ITKATIPLVGSPADIPYYYVPPQHP--VGPPGMIMG-
PvNF-YC4      130 TDIFDFLVDIVPRDEIKDDAAL--------VGATASGVPYYYPPIGQP-----AGMMIG-
PvNF-YC3       90 TDLFDFLITLVSNSDSHPLGVP---------TTTTMEVETIY------------------
PvNF-YC7       90 TDIFDFLITLVSNSDSHALGA----------TTTILEVETID------------------
PvNF-YC5      143 SDVFDFLVDTVPRDDTVTVGPQLLAG---IAPTPNQNAPYPYMSIPDQ-------HVAGP
NF-YC_Mouse   108 FDQFDFLIDIVPRDELKPPKRQEEVR---QSVTPAEPVQYYFTLAQQPTAVQVQGQQQPQ

PvNF-YC1      226 KPVMDPNMYAQQSHPY-MAPQMWPQPPDQR----------QPSPEH--------------
PvNF-YC6      228 NPVMDP--YAQQSHQYSMAPQMWPQPPEQQ----------QSSPDQ--------------
PvNF-YC2      229 KPVDQAALYSTQQPRPPMAFMPWPHTQPQP----------QHSPQQQTDS----------
PvNF-YC4      176 RPAVDPATGVYYVQPPSQAWQSVWQSAADDASYGGAGGAGAQGSIDGQS-----------
PvNF-YC3          ------------------------------------------------------------
PvNF-YC7          ------------------------------------------------------------
PvNF-YC5      193 PPPYAAPTNAPEDAANPSCSNSDPTPNEQD----------TSSSDDH-------------
NF-YC_Mouse   165 QTTSSTTTIQPGQIIIAQPQQGQTTPVTMQVGEGQQVQIVQAQPQGQAQQTQSGTGQTMQ

PvNF-YC1          ------------------------------------------------------------
PvNF-YC6          ------------------------------------------------------------
PvNF-YC2          ------------------------------------------------------------
PvNF-YC4          ------------------------------------------------------------
PvNF-YC3          ------------------------------------------------------------
PvNF-YC7          ------------------------------------------------------------
PvNF-YC5          ------------------------------------------------------------
NF-YC_Mouse   225 VMQQIITNTGETQQIPVQLNAAQLQYIRLAQPVSGTQVVQGQIQTLATNAQQITQTEVQQ

PvNF-YC1          ---------------------------------------------------
PvNF-YC6          ---------------------------------------------------
PvNF-YC2          ---------------------------------------------------
PvNF-YC4          ---------------------------------------------------
PvNF-YC3          ---------------------------------------------------
PvNF-YC7          ---------------------------------------------------
PvNF-YC5          ---------------------------------------------------
PvNF-YC_Mouse 285 GQEQFSQFTDGQQLYQIQQVTMPAGQDLAQPMFIQSANQPSDGQTPQVTGD
```

Figure S7A

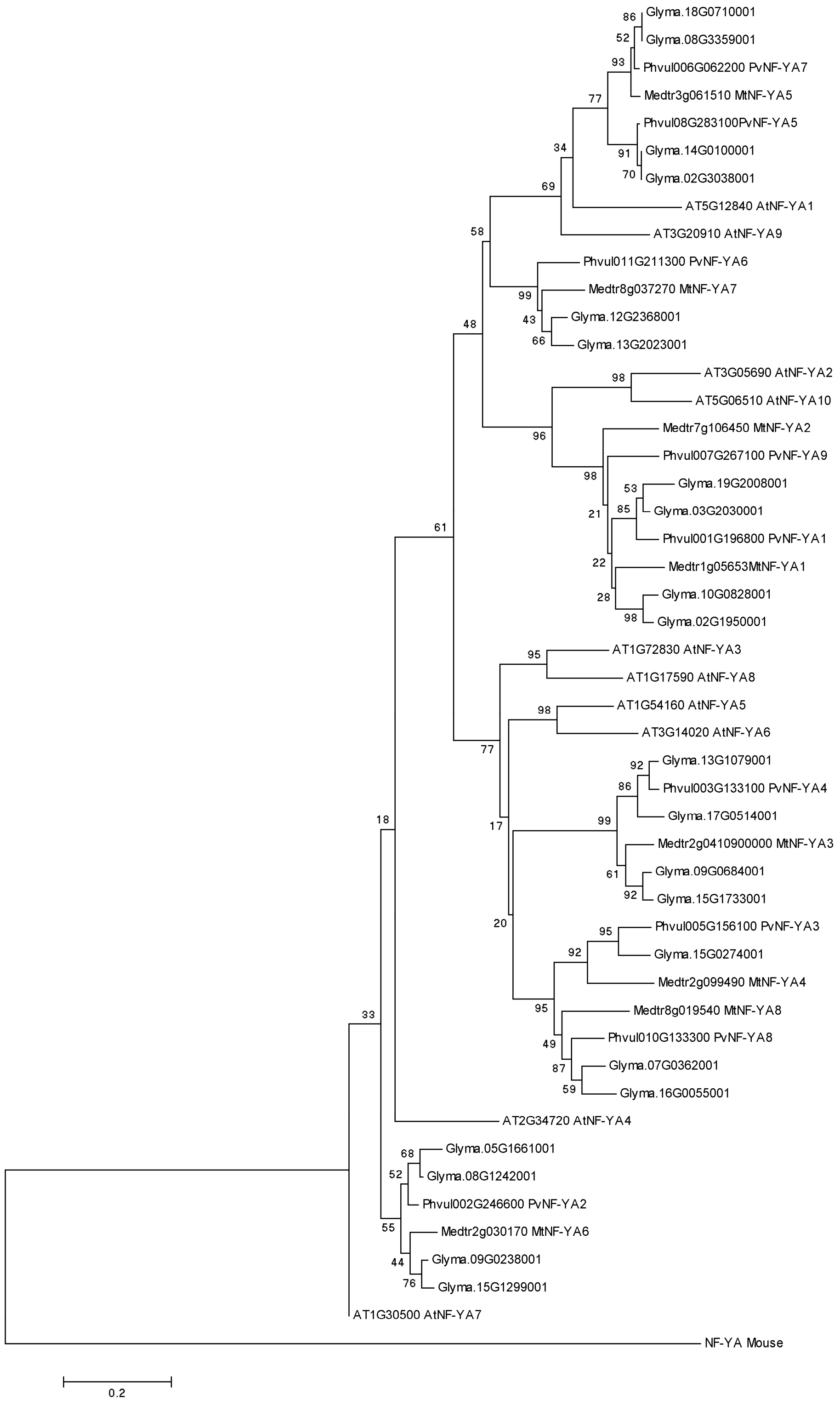
Glyma.18G0710001
Glyma.08G3359001
Phvul006G062200 PvNF-YA7
Medtr3g061510 MtNF-YA5
Phvul08G283100PvNF-YA5
Glyma.14G0100001
Glyma.02G3038001
AT5G12840 AtNF-YA1
AT3G20910 AtNF-YA9
Phvul011G211300 PvNF-YA6
Medtr8g037270 MtNF-YA7
Glyma.12G2368001
Glyma.13G2023001
AT3G05690 AtNF-YA2
AT5G06510 AtNF-YA10
Medtr7g106450 MtNF-YA2
Phvul007G267100 PvNF-YA9
Glyma.19G2008001
Glyma.03G2030001
Phvul001G196800 PvNF-YA1
Medtr1g05653MtNF-YA1
Glyma.10G0828001
Glyma.02G1950001
AT1G72830 AtNF-YA3
AT1G17590 AtNF-YA8
AT1G54160 AtNF-YA5
AT3G14020 AtNF-YA6
Glyma.13G1079001
Phvul003G133100 PvNF-YA4
Glyma.17G0514001
Medtr2g0410900000 MtNF-YA3
Glyma.09G0684001
Glyma.15G1733001
Phvul005G156100 PvNF-YA3
Glyma.15G0274001
Medtr2g099490 MtNF-YA4
Medtr8g019540 MtNF-YA8
Phvul010G133300 PvNF-YA8
Glyma.07G0362001
Glyma.16G0055001
AT2G34720 AtNF-YA4
Glyma.05G1661001
Glyma.08G1242001
Phvul002G246600 PvNF-YA2
Medtr2g030170 MtNF-YA6
Glyma.09G0238001
Glyma.15G1299001
AT1G30500 AtNF-YA7
NF-YA Mouse
0.2

Figure S7B

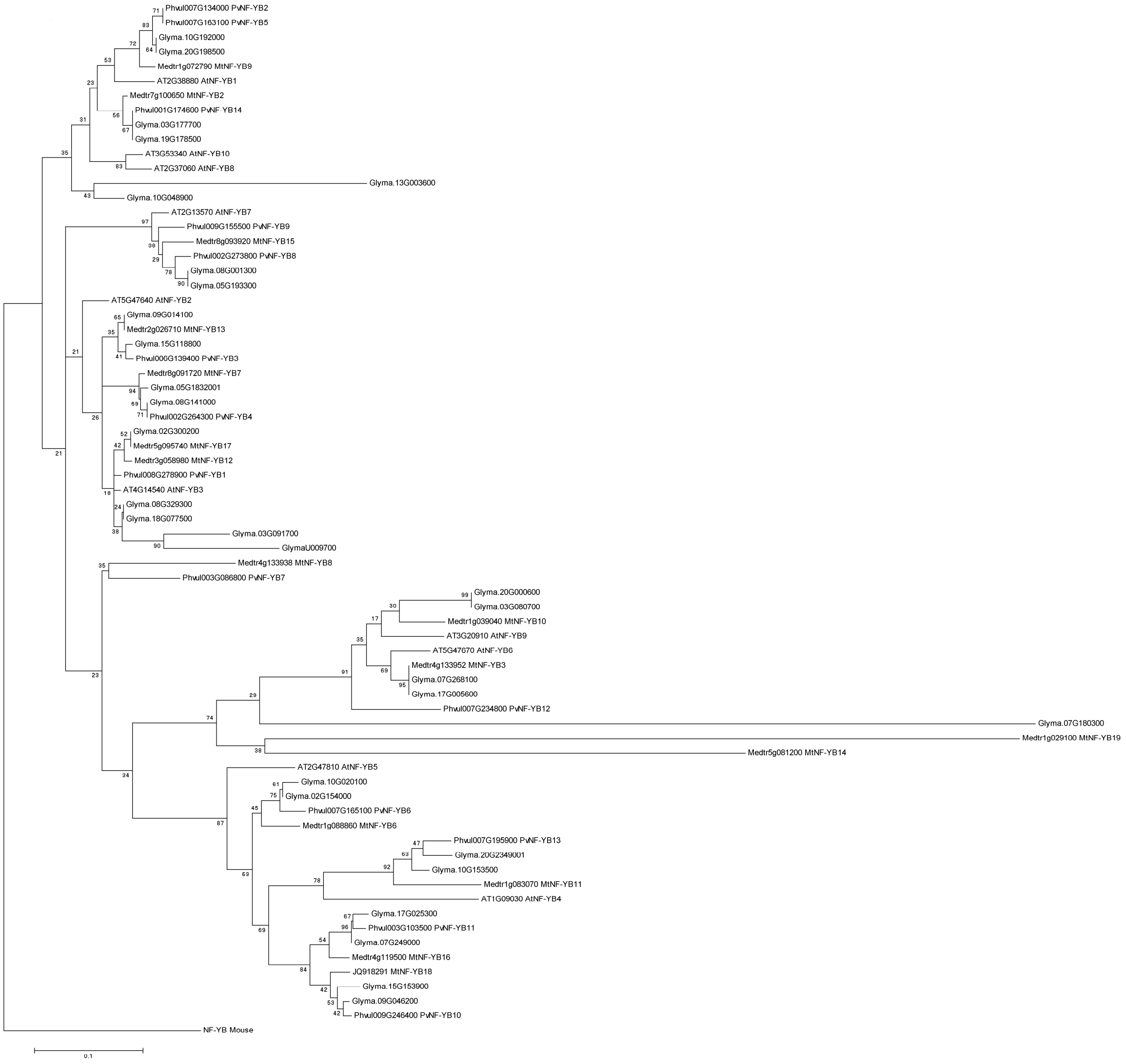
Phvul007G134000 PvNF-YB2
Phvul007G163100 PvNF-YB5
Glyma.10G192000
Glyma.20G198500
Medtr1g072790 MtNF-YB9
AT2G38880 AtNF-YB1
Medtr7g100650 MtNF-YB2
Phvul001G174600 PvNF-YB14
Glyma.03G177700
Glyma.19G178500
AT3G53340 AtNF-YB10
AT2G37060 AtNF-YB8
Glyma.13G003600
Glyma.10G048900
AT2G13570 AtNF-YB7
Phvul009G155500 PvNF-YB9
Medtr8g093920 MtNF-YB15
Phvul002G273800 PvNF-YB8
Glyma.08G001300
Glyma.05G193300
AT5G47640 AtNF-YB2
Glyma.09G014100
Medtr2g026710 MtNF-YB13
Glyma.15G118800
Phvul006G139400 PvNF-YB3
Medtr8g091720 MtNF-YB7
Glyma.05G1832001
Glyma.08G141000
Phvul002G264300 PvNF-YB4
Glyma.02G300200
Medtr5g095740 MtNF-YB17
Medtr3g058980 MtNF-YB12
Phvul008G278900 PvNF-YB1
AT4G14540 AtNF-YB3
Glyma.08G329300
Glyma.18G077500
Glyma.03G091700
GlymaU009700
Medtr4g133938 MtNF-YB8
Phvul003G086800 PvNF-YB7
Glyma.20G000600
Glyma.03G080700
Medtr1g039040 MtNF-YB10
AT3G20910 AtNF-YB9
AT5G47670 AtNF-YB6
Medtr4g133952 MtNF-YB3
Glyma.07G268100
Glyma.17G005600
Phvul007G234800 PvNF-YB12
Glyma.07G180300
Medtr1g029100 MtNF-YB19
Medtr5g081200 MtNF-YB14
AT2G47810 AtNF-YB5
Glyma.10G020100
Glyma.02G154000
Phvul007G165100 PvNF-YB6
Medtr1g088860 MtNF-YB6
Phvul007G195900 PvNF-YB13
Glyma.20G2349001
Glyma.10G153500
Medtr1g083070 MtNF-YB11
AT1G09030 AtNF-YB4
Glyma.17G025300
Phvul003G103500 PvNF-YB11
Glyma.07G249000
Medtr4g119500 MtNF-YB16
JQ918291 MtNF-YB18
Glyma.15G153900
Glyma.09G046200
Phvul009G246400 PvNF-YB10
NF-YB Mouse
0.1

Figure S7C

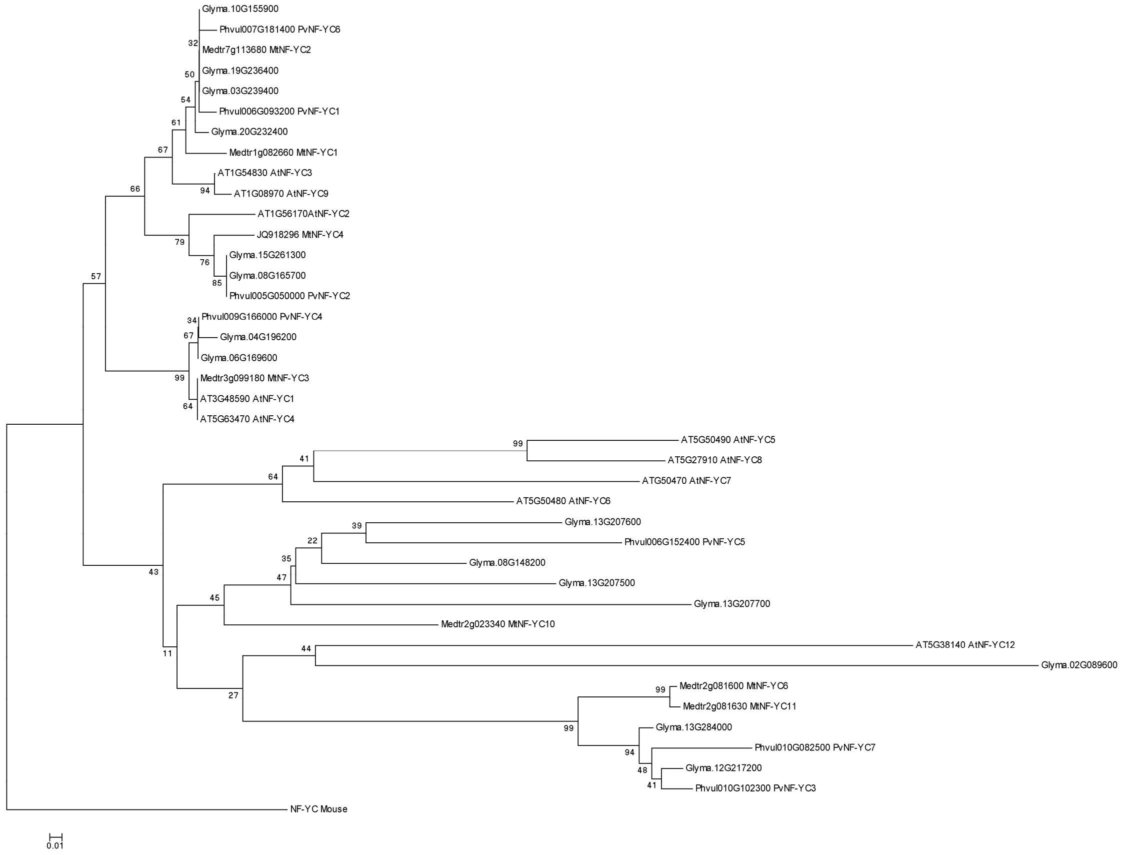
Glyma.10G155900
Phvul007G181400 PvNF-YC6
Medtr7g113680 MtNF-YC2
Glyma.19G236400
Glyma.03G239400
Phvul006G093200 PvNF-YC1
Glyma.20G232400
Medtr1g082660 MtNF-YC1
AT1G54830 AtNF-YC3
AT1G08970 AtNF-YC9
AT1G56170AtNF-YC2
JQ918296 MtNF-YC4
Glyma.15G261300
Glyma.08G165700
Phvul005G050000 PvNF-YC2
Phvul009G166000 PvNF-YC4
Glyma.04G196200
Glyma.06G169600
Medtr3g099180 MtNF-YC3
AT3G48590 AtNF-YC1
AT5G63470 AtNF-YC4
AT5G50490 AtNF-YC5
AT5G27910 AtNF-YC8
ATG50470 AtNF-YC7
AT5G50480 AtNF-YC6
Glyma.13G207600
Phvul006G152400 PvNF-YC5
Glyma.08G148200
Glyma.13G207500
Glyma.13G207700
Medtr2g023340 MtNF-YC10
AT5G38140 AtNF-YC12
Glyma.02G089600
Medtr2g081600 MtNF-YC6
Medtr2g081630 MtNF-YC11
Glyma.13G284000
Phvul010G082500 PvNF-YC7
Glyma.12G217200
Phvul010G102300 PvNF-YC3
NF-YC Mouse
0.01

Figure S8A

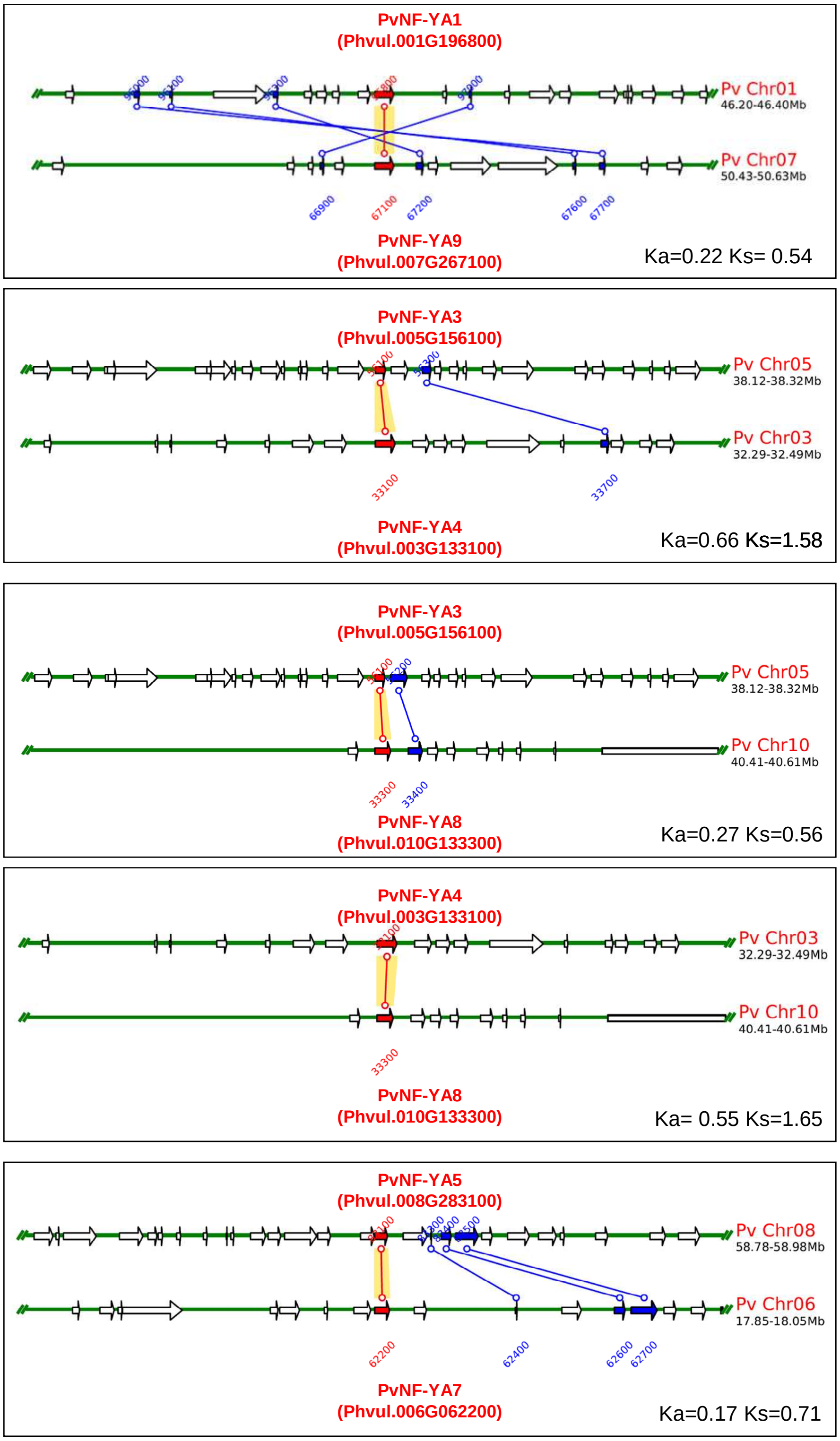
PvNF-YA1
(Phvul.001G196800)
Pv Chr01
46.20-46.40Mb
Pv Chr07
50.43-50.63Mb
66900
67100
67200
67600
67700
PvNF-YA9
(Phvul.007G267100)
Ka=0.22 Ks= 0.54
PvNF-YA3
(Phvul.005G156100)
Pv Chr05
38.12-38.32Mb
Pv Chr03
32.29-32.49Mb
33100
33700
PvNF-YA4
(Phvul.003G133100)
Ka=0.66 Ks=1.58
PvNF-YA3
(Phvul.005G156100)
Pv Chr05
38.12-38.32Mb
Pv Chr10
40.41-40.61Mb
33300
33400
PvNF-YA8
(Phvul.010G133300)
Ka=0.27 Ks=0.56
PvNF-YA4
(Phvul.003G133100)
Pv Chr03
32.29-32.49Mb
Pv Chr10
40.41-40.61Mb
33300
PvNF-YA8
(Phvul.010G133300)
Ka= 0.55 Ks=1.65
PvNF-YA5
(Phvul.008G283100)
Pv Chr08
58.78-58.98Mb
Pv Chr06
17.85-18.05Mb
62200
62400
62600
62700
PvNF-YA7
(Phvul.006G062200)
Ka=0.17 Ks=0.71

Figure S8B

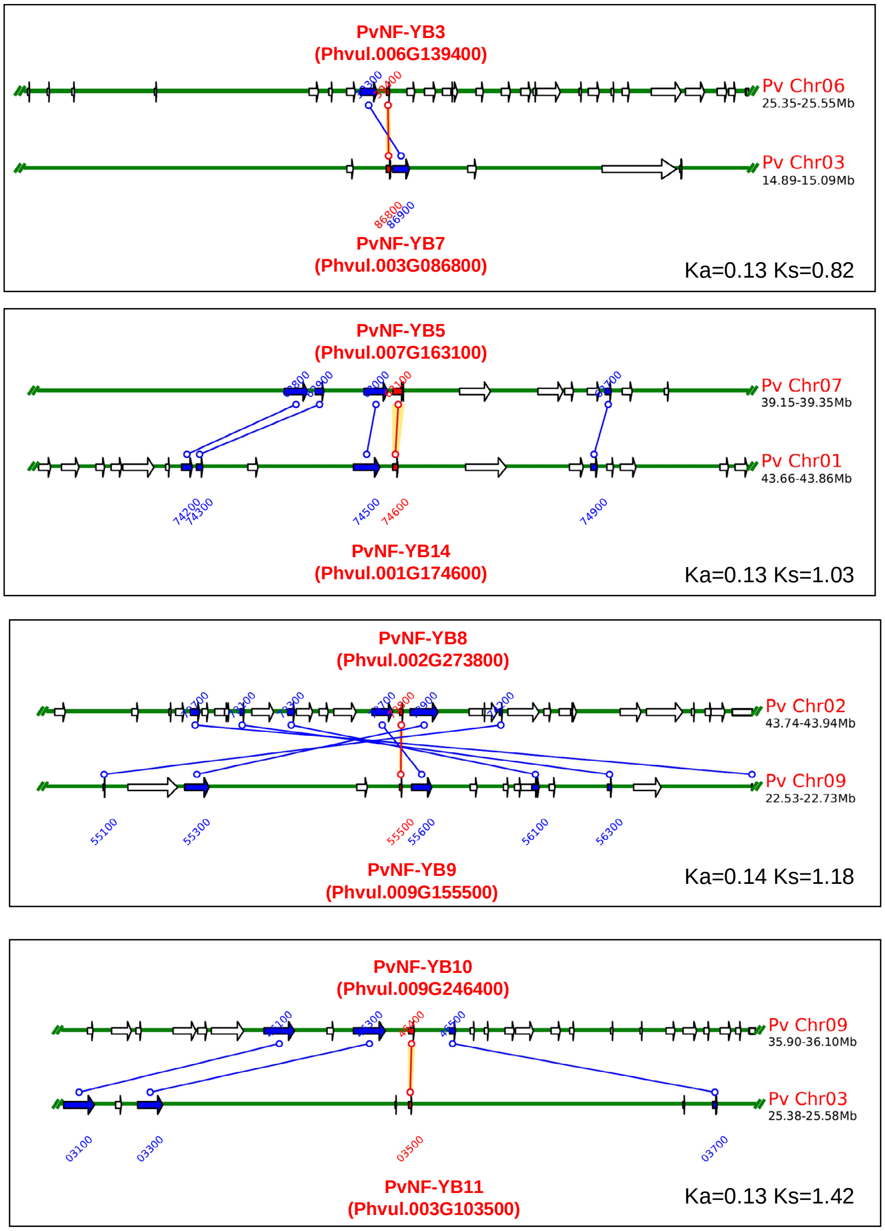
PvNF-YB3
(Phvul.006G139400)
Pv Chr06
25.35-25.55Mb
Pv Chr03
14.89-15.09Mb
86800
86900
PvNF-YB7
(Phvul.003G086800)
Ka=0.13 Ks=0.82
PvNF-YB5
(Phvul.007G163100)
Pv Chr07
39.15-39.35Mb
Pv Chr01
43.66-43.86Mb
74200
74300
74500
74600
74900
PvNF-YB14
(Phvul.001G174600)
Ka=0.13 Ks=1.03
PvNF-YB8
(Phvul.002G273800)
Pv Chr02
43.74-43.94Mb
Pv Chr09
22.53-22.73Mb
55100
55300
55500
55600
56100
56300
PvNF-YB9
(Phvul.009G155500)
Ka=0.14 Ks=1.18
PvNF-YB10
(Phvul.009G246400)
Pv Chr09
35.90-36.10Mb
Pv Chr03
25.38-25.58Mb
03100
03300
03500
03700
PvNF-YB11
(Phvul.003G103500)
Ka=0.13 Ks=1.42

Figure S8C

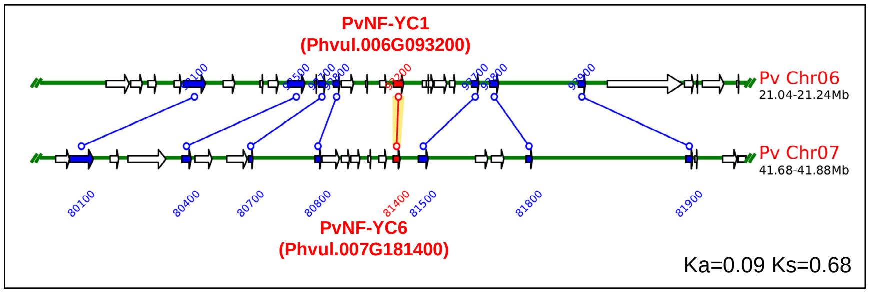
PvNF-YC1
(Phvul.006G093200)
Pv Chr06
21.04-21.24Mb
Pv Chr07
41.68-41.88Mb
80100
80400
80700
80800
81400
81500
81800
81900
PvNF-YC6
(Phvul.007G181400)
Ka=0.09 Ks=0.68
