# Supplementary material for: Annotation, phylogeny and expression analysis of the nuclear factor Y gene families in common bean (Phaseolus vulgaris)
Source: Front Plant Sci. 2015 Jan 14;5:761. doi: 10.3389/fpls.2014.00761 (PMC4294137; doi:10.3389/fpls.2014.00761)
Supplement: Supplementary file 2 [file Table1.DOC]

| ***P. vulgaris* NF-YA family**  **Table S1. Primers used for quantitative reverse-transcriptase polymerase chain reaction analysis** | | ***P. vulgaris* NF-YB family** | | | ***P. vulgaris* NF-YC family** | | |  | |
| --- | --- | --- | --- | --- | --- | --- | --- | --- | --- |
| **Primer name** | **Sequence (5**'**-3**'**)** | | **Primer name** | **Sequence (5**'**-3**'**)** | | **Primer name** | **Sequence (5**'**-3**'**)** | |  |
| **qNF-YA1 F^a^** | TCATTTTGGATCTTCTGTGCACAC | | **qNF-YB1 F** | GTTCCATCAAGTGAGTGCCG | | **qNF-YC1 F^b^** | GCAGGGCTATATCGGTCTTTTC | |  |
| **qNF-YA1 R^a^** | GCTTCTTCATCTGGTCTCATAAAGG | | **qNF-YB1 R** | AGGAGTGGCAAATAGAGGACC | | **qNF-YC1 R^b^** | GAGTAACATTTTGGATTCAATG | |  |
| **qNF-YA2 F** | GTGTGGTAGGTACGTTAGGATG | | **qNF-YB2 F** | GACATGGATGGAAAGAATGGTGTAA | | **qNF-YC2 F^b^** | AGCATTCACCCCAAAAACAAACAG | |  |
| **qNF-YA2 R** | CTTCTGACAATCTCTAATCTCTCTACAG | | **qNF-YB2 R** | CCACCCCAACACACGTTTT | | **qNF-YC2 R^b^** | GAGTGCAAAAATCCTCAAACAATCTG | |  |
| **qNF-YA3 F** | CATACATCTGATAAGGGTGGTGG | | **qNF-YB3 F** | GTTTGTAGGTGGTGGCGTTT | | **qNF-YC3 F^b^** | ACGGGATAAGAAACAGTAGAGAAC | |  |
| **qNF-YA3 R** | CCAAACAAACAGCAATAAATAGCAAC | | **qNF-YB3 R** | CATTGCCTCCATTCGTGTTACC | | **qNF-YC3 R^b^** | CGGTATTAGTTAGAAGGGTTATTGTG | |  |
| **qNF-YA4 F** | ATTTCAGGCAGCCCGAACA | | **qNF-YB4 F** | ACGTTATAGGGTTGAATGGTAGAT | | **qNF-YC4 F** | CAGGAGTTATGAAGACGCAGAAC | |  |
| **qNF-YA4 R** | AAGGATGATTTGCCAGACCAGT | | **qNF-YB4 R** | AAGAAAGCGTGTGAAATGTAGC | | **qNF-YC4 R** | CCCACAGAAGGATCAATTTTTAGTTT | |  |
| **qNF-YA5 F** | GCGTCTTGCTATTCAGTAAACCAC | | **qNF-YB5 F** | TGTACCCCTTGGCATATTGAATG | | **qNF-YC5 F** | TCATCCAGTGTTTGCAGGCATA | |  |
| **qNF-YA5R** | CCATATGATGATTAGCATAAACACAGCC | | **qNF-YB5 R** | TTGATGGAATAAAACAAGGACTGATCC | | **qNF-YC5 R** | AAGGGTTTGCAGCTCAGGATA | |  |
| **qNF-YA6 F** | GGCTTTCAAATAGCACTTCCCTC | | **qNF-YB6 F** | TGAGAGAGCAAACCAAAATAAGGG | | **qNF-YC6 F** | AACAATCATCTCCAGACCAGTAGC | |  |
| **qNF-YA6 R** | ATAGAGTACACCATGCCACTGC | | **qNF-YB6 R** | ACCTTGAACATCTGAAATGTGAGG | | **qNF-YC6 R** | ACTGAAATGAACAGTCCAACAAAAAGG | |  |
| **qNF-YA7 F** | TCAGGTGAAAGAGTGGAGGAAG | | **qNF-YB7 F** | GATGGAAGGGGAGAAAACTGC | | **qNF-YC7 F** | GCCATCCAAGGCAAAAGGAAG | |  |
| **qNF-YA7 R** | CCACAAACACAAGAACAAAGCCA | | **qNF-YB7 R** | CCCTATGTGCATCCACAATTACC | | **qNF-YC7 R** | GTTTCAACCTCCAAAATAGTGGTGG | |  |
| **qNF-YA8 F** | GGAAACCAACACCGTCTATCAG | | **qNF-YB8 F** | AGAGGGAGAAGAGAAAGACCATC | |  |  | |  |
| **qNF-YA8 R** | GGATGACTTCCCCATGTAACAC | | **qNF-YB8 R** | GTGTTGTTGTAGCCTTTGTTCAGA | |  |  | |  |
| **qNF-YA9 F** | AGGTTTCGATGGCTTTTCTGT | | **qNF-YB9 F** | TCGTTCTGAACAAAGGCTACAAC | |  |  | |  |
| **qNF-YA9 R** | CATCTAGTGGGCATGGTGATAC | | **qNF-YB9 R** | CTGAATTGAGTTTGGAGAGAAGGG | |  |  | |  |
|  |  | | **qNF-YB10 F** | GGAGAAAGACAGTGAATGGTGATG | |  |  | |  |
|  |  | | **qNF-YB10 R** | GGATTTTCGTCCTTGATTTCCTTCTC | |  |  | |  |
|  |  | | **qNF-YB11 F** | TGCAGAGCCATTGAGAAGG | |  |  | |  |
|  |  | | **qNF-YB11 R** | ATGGTTGTTCTAGCTTGAGGG | |  |  | |  |
|  |  | | **qNF-YB12 F** | CCTTATGCTAATCCACCAAAACCT | |  |  | |  |
|  |  | | **qNF-YB12 R** | ACTGAAGAAAACACAACACAGGAAAG | |  |  | |  |
|  |  | | **qNF-YB13 F** | GGACTTCCCCTTCTACCTTCTTTA | |  |  | |  |
|  |  | | **qNF-YB13 R** | GCCTTGCCCAGTTTTATCTTCAAC | |  |  | |  |
|  |  | | **qNF-YB14 F** | AGGTCCAGAGTAGGTGTCAAG | |  |  | |  |
|  |  | | **qNF-YB14 R** | ACAAGAACAAAGATCTCCTATGTCAGT | |  |  | |  |

^a^ Primers decsribed in Battaglia et al, 2014. ^b^ Primers described in Zanetti et al., 2010.
