# Supplementary material for: Annotation, phylogeny and expression analysis of the nuclear factor Y gene families in common bean (Phaseolus vulgaris)
Source: Front Plant Sci. 2015 Jan 14;5:761. doi: 10.3389/fpls.2014.00761 (PMC4294137; doi:10.3389/fpls.2014.00761)
Supplement: Supplementary file 4 [file Table3.DOC]

**Table S3**. Identity matrix of the NF-YA family for full length proteins.

**Amino-acid identity**

PvNF-YA1 PvNF-YA9 Pv NF-YA3 PvNF-YA8 PvNF-Y4 PvNF-YA5 PvNF-YA7 PvNF-YA6 Pv NF-YA2 NF-YA_ Mouse

PvNF-YA1 100.00 **63.23** 23.91 23.38 31.22 21.60 21.59 23.85 27.18 21.95

PvNF-YA9 **63.23** 100.00 27.49 26.04 28.96 25.90 22.60 27.24 29.02 26.94

PvNF-YA3 23.91 27.49 100.00 **61.48** 39.80 22.09 19.01 25.91 34.55 24.00

PvNF-YA8 23.38 26.04 **61.48** 100.00 41.44 25.44 21.43 25.86 36.41 27.00

PvNF-YA4 31.22 28.96 39.80 41.44 100.00 31.25 28.38 30.69 41.42 33.12

PvNF-YA5 21.60 25.90 22.09 25.44 31.25 100.00 **71.48** 43.67 30.56 23.50

PvNF-YA7 21.59 22.60 19.01 21.43 28.38 **71.48** 100.00 43.96 30.39 21.24

PvNF-YA6 23.85 27.24 25.91 25.86 30.69 43.67 43.96 100.00 40.62 23.61

PvNF-YA2 27.18 29.02 34.55 36.41 41.42 30.56 30.39 40.62 100.00 26.15

NF-YA_Mouse 21.95 26.94 24.00 27.00 33.12 23.50 21.24 23.61 26.15 100.00
