# Supplementary material for: Annotation, phylogeny and expression analysis of the nuclear factor Y gene families in common bean (Phaseolus vulgaris)
Source: Front Plant Sci. 2015 Jan 14;5:761. doi: 10.3389/fpls.2014.00761 (PMC4294137; doi:10.3389/fpls.2014.00761)
Supplement: Supplementary file 5 [file Table4.DOC]

**Table S4**. Identity matrix of the NF-YB family for full length proteins.

Amino-acid identity

PvNF-YB12 PvNF-YB13 PvNF-YB110 PvNF-YB11 PvNF-YB6 PvNF-YB8 PvNF-YB9 PvNF-YB3 PvNF-YB7 PvNF-YB1 PvNF-YB4 PvNF-YB2 PvNF-YB5 PvNF-YB14 NF-Y Mouse

PvNF-YB12 100.00 46.21 53.12 51.28 50.85 39.38 37.89 46.85 53.45 42.41 41.72 40.27 40.27 40.51 33.96

PvNF-YB13 46.21 100.00 66.67 62.39 67.52 47.86 47.89 51.15 60.00 44.29 45.45 45.04 45.04 39.57 39.72

PvNF-YB10 53.12 66.67 100.00 **80.00** 69.09 55.45 59.62 60.00 65.00 58.10 58.10 56.88 56.88 56.19 54.55

PvNF-YB11 51.28 62.39 **80.00** 100.00 58.67 46.71 49.32 53.28 59.69 49.64 48.92 51.45 51.45 48.15 40.27

PvNF-YB6 50.85 67.52 69.09 58.67 100.00 52.98 55.56 54.74 61.24 53.28 56.12 52.90 52.90 53.33 43.62

PvNF-YB8 39.38 47.86 55.45 46.71 52.98 100.00 **76.78** 46.67 59.09 50.00 45.73 42.77 42.77 47.19 36.14

PvNF-YB9 37.89 47.89 59.62 49.32 55.56 **76.78** 100.00 48.28 59.38 47.51 44.39 42.77 42.77 45.20 38.34

PvNF-YB3 46.85 51.15 60.00 53.28 54.74 46.67 48.28 100.00 79.55 69.82 55.00 51.88 51.88 54.60 44.10

PvNF-YB7 53.45 60.00 65.00 59.69 61.24 59.09 59.38 79.55 100.00 67.42 63.64 60.77 60.77 63.57 47.73

PvNF-YB1 42.41 44.29 58.10 49.64 53.28 50.00 47.51 69.82 67.42 100.00 54.01 53.57 53.57 54.14 42.70

PvNF-YB4 41.72 45.45 58.10 48.92 56.12 45.73 44.39 55.00 63.64 54.01 100.00 46.15 46.15 48.35 43.65

PvNF-YB2 40.27 45.04 56.88 51.45 52.90 42.77 42.77 51.88 60.77 53.57 46.15 100.00 100.00 70.66 45.03

PvNF-YB5 40.27 45.04 56.88 51.45 52.90 42.77 42.77 51.88 60.77 53.57 46.15 100.00 100.00 70.66 45.03

PvNF-YB14 40.51 39.57 56.19 48.15 53.33 47.19 45.20 54.60 63.57 54.14 48.35 70.66 70.66 100.00 44.63

NF-YB_Mouse 33.96 39.72 54.55 40.27 43.62 36.14 38.34 44.10 47.73 42.70 43.65 45.03 45.03 44.63 100.00
