# Supplementary material for: Annotation, phylogeny and expression analysis of the nuclear factor Y gene families in common bean (Phaseolus vulgaris)
Source: Front Plant Sci. 2015 Jan 14;5:761. doi: 10.3389/fpls.2014.00761 (PMC4294137; doi:10.3389/fpls.2014.00761)
Supplement: Supplementary file 6 [file Table5.DOC]

**Table S5**. Identity matrix of the NF-YC family for full length proteins.

Amino-acid identity

PvNF-YC1 PvNF-YC6 PvNF-YC2 PvNF-YC4 PvNF-YC3 PvNF-YC7 PvNF-YC5 NF-YC_Mouse

PvNF-YC1 100.00 **85.60** 59.13 56.73 46.28 45.00 39.56 41.54

PvNF-YC6 **85.60** 100.00 58.10 56.04 44.63 45.00 40.44 40.51

PvNF-YC2 59.13 58.10 100.00 57.75 44.63 42.50 37.78 42.42

PvNF-YC4 56.73 56.04 57.75 100.00 49.59 48.33 38.76 45.03

PvNF-YC3 46.28 44.63 44.63 49.59 100.00 **86.78** 42.98 43.81

PvNF-YC7 45.00 45.00 42.50 48.33 **86.78** 100.00 40.83 40.38

PvNF-YC5 39.56 40.44 37.78 38.76 42.98 40.83 100.00 36.13

NF-YC_Mouse 41.54 40.51 42.42 45.03 43.81 40.38 36.13 100.00
